# The *Arabidopsis* HEI10 Is a New ZMM Protein Related to Zip3

**DOI:** 10.1371/journal.pgen.1002799

**Published:** 2012-07-26

**Authors:** Liudmila Chelysheva, Daniel Vezon, Aurélie Chambon, Ghislaine Gendrot, Lucie Pereira, Afef Lemhemdi, Nathalie Vrielynck, Sylvia Le Guin, Maria Novatchkova, Mathilde Grelon

**Affiliations:** 1 INRA, UMR1318, Institut Jean-Pierre Bourgin, RD10, Versailles, France; 2 AgroParisTech, Institut Jean-Pierre Bourgin, RD10, Versailles, France; 3 Université de Lyon, Ecole Normale Supérieure de Lyon, Université Lyon 1, IFR128 BioSciences Lyon Gerland, Unité Reproduction et Développement des Plantes, Lyon, France; 4 Research Institute of Molecular Pathology, Vienna, Austria; 5 Institute of Molecular Biotechnology, Austrian Academy of Sciences, Vienna, Austria; University of Birmingham, United Kingdom

## Abstract

In numerous species, the formation of meiotic crossovers is largely under the control of a group of proteins known as ZMM. Here, we identified a new ZMM protein, HEI10, a RING finger-containing protein that is well conserved among species. We show that HEI10 is structurally and functionally related to the yeast Zip3 ZMM and that it is absolutely required for class I crossover (CO) formation in *Arabidopsis thaliana*. Furthermore, we show that it is present as numerous foci on the chromosome axes and the synaptonemal complex central element until pachytene. Then, from pachytene to diakinesis, HEI10 is retained at a limited number of sites that correspond to class I COs, where it co-localises with MLH1. Assuming that HEI10 early staining represents an early selection of recombination intermediates to be channelled into the ZMM pathway, HEI10 would therefore draw a continuity between early chosen recombination intermediates and final class I COs.

## Author Summary

During meiosis two successive chromosomal divisions follow a single S phase, resulting in the formation of four haploid cells, each with half of the parental genetic material. This ploidy reduction occurs during the first meiotic division, when homologous chromosomes (paternal and maternal) are separated from each other. For this to happen, homologous chromosomes associate in bivalents, where each chromosome is linked to its homologue by chiasmata. These chiasmata reflect the formation of crossovers (COs), one of the manifestations of the exchange of genetic material occurring during homologous recombination. In most species, the final number of COs represents only a small proportion of all meiotic recombination events (4% in *Arabidopsis thaliana*). The mechanisms that drive the choice of recombination intermediates that will mature into COs are still unknown. In this study, we identified the HEI10 protein that is present as numerous foci on chromosome axes during early meiotic prophase and is retained until the end of prophase at a limited number of sites corresponding to COs. We also showed that HEI10 is necessary for the formation of most of the COs. HEI10 is therefore a good candidate for a ZMM protein involved in generating continuity between chosen early recombination intermediates and final COs.

## Introduction

From a diploid mother cell, meiosis generates four haploid products from which gametes differentiate. This ploidy reduction is a direct consequence of two rounds of chromosomal segregation (meiosis I and meiosis II) following a single S phase. The first meiotic division separates homologous chromosomes from each other while meiosis II separates sister chromatids. Accurate segregation of homologous chromosomes at the first meiotic division is dependent on the formation of physical connections, known as chiasmata, between homologous chromosome pairs. Chiasmata arise from homologous recombination (HR) during prophase I of meiosis and are the physical manifestation of genetic crossovers (COs).

Homologous recombination is initiated by programmed DNA double-strand breaks (DSB) catalyzed by the Spo11 protein [1]. DSB formation is followed by recombinational repair, facilitated by the RecA homologs RAD51 and DMC1. RAD51 and DMC1 coat the 3′-single-stranded tails and promote single end invasions (SEIs) of homologous intact duplexes that are used for DNA repair. At least some of these events happen using the homologous chromosome as a template and result in either CO or non crossover (NCO) products [2]. CO events are highly regulated both within and among chromosomes. At least one obligatory CO is required for each pair of chromosomes, regardless of their size. When multiple COs are present, they are more widely spaced than predicted for a random distribution. This phenomenon is known as positive interference [3]. At least two kinds of COs can coexist in *Saccharomyces cerevisiae*, mammals and plants. Class I COs are interference-sensitive, and their formation is dependent on a group of proteins initially called ZMM proteins (Zip1, Msh4, Msh5 and Mer3) but also containing Zip2, Zip3, Zip4 and Spo16 [4]. In plants, Class I COs depend on *AtMSH4*, *AtMSH5*, *AtMER3*, *AtZIP4*, *AtSHOC1/ZIP2* and *PTD* (reviewed in [5]). Class II COs, however, are interference insensitive and lead to randomly distributed COs, most of which require the Mus81 and Mms4 proteins at least in *S. cerevisiae* and *Arabidopsis* [6], [7], [8].

In budding yeast, ZMM proteins may load collaboratively and appear to specifically mark CO sites because the number of ZMM foci, including Msh4, Zip2, Zip3 and Zip4, is similar to CO number and they are distributed non-randomly just like interference-sensitive COs [9]. In contrast to its *S. cerevisiae* counterparts, the dynamic of ZMM loading is different in others organisms. For example, MSH4/MSH5 foci outnumber COs in mouse and *A. thaliana* [10], [11], [12], [13]. At early stages they co-localise with DMC1/RAD51 foci on meiotic chromosomes and mark numerous interhomologue recombinational interactions, most of which do not result in COs [10], [11], [14], [15], [16]. As meiosis progresses, a subset of MSH4/MSH5 foci may be stabilised by interactions with MLH1–MLH3 heterodimers. Mlh1-Mlh3 are eukaryotic homologues of *Escherichia coli* MutL genes, they are essential for wild-type levels of crossing over in budding yeast, mammals and plants [17], [18], [19], [20], [21]. These sites eventually become the COs, while the fate of the additional MSH4–MSH5 sites that were not stabilised by MLH1–MLH3 is unknown.

In yeast, ZMM also interact in the assembly of the synaptonemal complex (SC), an elaborate proteinaceous structure that holds homologues close together along their lengths [22]. The SC is a tripartite structure consisting of two parallel axial elements (AE), each representing one pair of sister chromatids, and an intervening central element (CE). A major component of the CE is a long coiled-coil protein (Zip1 in budding yeast, ZYP1 in *Arabidopsis*, SYCP1 in mammals, ZEP1 in rice), whose polymerization forms the transverse filament [23]. In budding yeast, ZMM proteins Zip2, Zip3, Zip4/Spo22, and Spo16 form Synapsis Initiation Complexes (SIC), which are required for polymerization of Zip1 along the lengths of chromosomes [24], [25], [26], [27]. Zip3 acts upstream of the other SIC components and is believed to facilitate and/or stabilise the localisation of Zip2, Zip4, and Spo16 to chromosomes. The fact that all the SIC components are necessary for class I COs, and that the number of SICs corresponds with the number of COs strongly suggests that, in *S. cerevisiae*, synapsis proceeds from class I CO sites. The ZMM functions required for normal SC assembly have also been observed in mice, where *Msh4* and *Msh5* are required for proper chromosome synapsis in both male and female meiosis [12], [27], [28]. In contrast to the situation in yeast and mice, the role of ZMM in SC assembly in plants appears minor. Minor defects in homolog synapsis were reported for the *Atmsh4* and *Atmer3* mutants in *Arabidopsis* and for a *mer3* mutant in rice [10], [29], [30], [31]. Furthermore, in contrast to other species [11], [32], chromosome synapsis proceeds normally in the *Atmsh5* and *Atzip4* mutants.

The current model of ZMM function during meiotic recombination proposes that these proteins act at CO-designated sites to stabilise early recombination intermediates. The Mer3 helicase is thought to act early after the D loop formation to stimulate the heteroduplex extension, stabilising nascent D loop structures [33]. Msh4 and Msh5, known to form a heterodimer, could act as a DNA clamp holding homologous chromosomes together, thereby stabilising the Holliday junction and facilitating CO formation [34]. SHOC1/ZIP2, which was recently shown to interact with the *Arabidopsis* ERCC1-like protein PTD, forms an XPF-ERCC1-related heterodimer, and could also be involved in double Holliday junction stabilisation [35], [36]. On the other hand no direct link to DNA metabolism has been found for the Zip3 proteins. These RING-domain-containing proteins [37] have a SUMO (small ubiquitin-related modifier) ligase activity necessary at least for SC assembly and spore viability [38].

In this paper we report the identification of a new ZMM protein in *Arabidopsis*. It is similar to the mammalian protein HEI10 (for Enhancer of cell Invasion N°10) also called CCNB1IP1 (for CyCliN B1 Interacting Protein 1), a RING-domain-containing protein shown to have an E3 ubiquitin ligase activity *in vitro*, and previously shown to be involved in meiotic recombination in mammals [39]. Here we show that HEI10 is necessary for class I CO formation and is structurally and functionally related to Zip3. We also show that HEI10 is loaded early during prophase on a large number of recombination sites. Then, while recombination progresses, HEI10 remains at sites which correspond to class I COs where it co-localises with MLH1 until the end of recombination.

## Results

### Identification and molecular characterisation of a *hei10* allelic series

In a screen for *A. thaliana* meiotic mutants, we isolated five mutant lines (EHH9, EHH69, EQO124, EVM265 and Salk_014624) allelic for disruption in At1g53490 (see Materials and Methods). Because of sequence similarity results (see below), we named this gene *Arabidopsis thaliana HEI10* and the corresponding mutations *hei10-1* (EQO124, in Ws-4 ecotype), *hei10-2* (Salk_014624, seed stock N514624, in Col-0 ecotype), *hei10-3* (EHH69, Ws-4 ecotype), *hei10-4* (EHH9, Ws-4 ecotype), and *hei10-5* (EVM265, Ws-4 ecotype).

**Figure 1:**
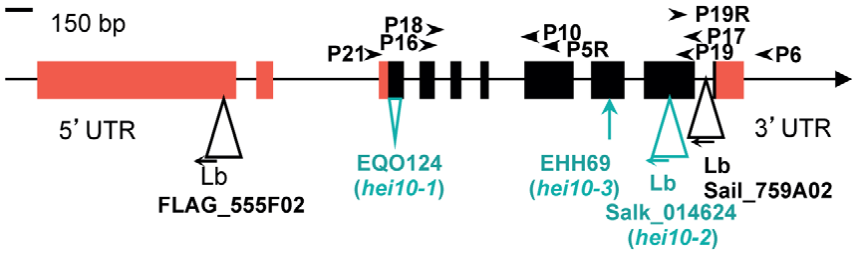
Characterisation of mutations in the HEI10 genomic region. Arrows indicate the orientation of the open reading frame, arrowheads indicate primer locations, and exons are shown as boxes (red: UTR, black: CDS). The mutations linked to a meiotic defect are shown in green, while insertions that do not induce a detectable phenotype are indicated in black. For T-DNA inserts, the FST name is given, together with the orientation of the sequenced flanking border. Lb: T-DNA left border.

Sequencing of At1g53490 in the *hei10-1* mutant line revealed a 44 pb deletion in the third exon of the gene, corresponding to the nucleotides just downstream of the start codon (Figure 1). Reverse-transcriptase PCR (RT-PCR) on flower bud cDNA from mutant plants showed that this deletion is associated with the production of modified transcripts corresponding to abnormal splicing variants of the third intron of *HEI10* (Figures S1 and S2A). These transcripts encode proteins truncated after amino acids 2 to 5 (Figure S2), showing that the *hei10-1* allele corresponds to a null mutation. This is also likely to be the case for *hei10-3* in which a single nucleotide insertion occurred in the eighth exon of At1g53490, leading to a premature stop codon at amino acid 227, and for alleles *hei10-4* and *hei10-5* in which the whole At1g53490 genomic region was deleted (see Materials and Methods, and Figure S3 and 4). In *hei10-2*, the T-DNA is inserted in the ninth exon of At1g53490 (Figure 1). Expression studies showed that the region 5′ to the T-DNA is transcribed but the transcript is interrupted by the T-DNA insertion (Figure S1A). This allele is therefore predicted to lead to the production of a truncated protein of 279 amino acids (instead of 304 in the wild type).

RT-PCR studies on cDNA isolated from different organs from wild type showed that the *Arabidopsis thaliana HEI10* is expressed mostly in flower buds and roots. The cDNA was not detectable in leaves (Figure S5).

### HEI10 is necessary for normal fertility in *Arabidopsis*

The *hei10-1* to *hei10-5* mutants all displayed the same phenotype: normal vegetative growth but fertility defects (Figure S6). The mean seed number per silique was 6.34 seeds per silique for *hei10-1* (n = 1,524 siliques) and 6.66 seeds/silique for *hei10-2* (n = 1,324) whereas wild-type siliques contained on average 63 and 71 seeds per silique for Ws (*hei10-1* ecotype) and Col0 (*hei10-2* ecotype), respectively (n = 50). We examined the reproductive development of these mutants and found that the *hei10* mutants were sterile due to abortion of male and female gametophytes (not shown). Comparison of the early stages of microsporogenesis revealed no difference between wild-type and mutant plants, with round pollen mother cells (PMCs) found within the anther locules (not shown). In wild-type anthers, these cells underwent two meiotic divisions to produce a characteristic tetrad of microspores (Figure S6). Meiotic products were also detected in mutant plants, but they lacked the regular tetrahedral structure and were either asymmetric tetrads or “polyads” containing more than four products (Figure S6), suggesting that the meiotic program is disturbed in *hei10* mutants.

### HEI10 is necessary for normal CO levels in *Arabidopsis*

**Figure 2:**
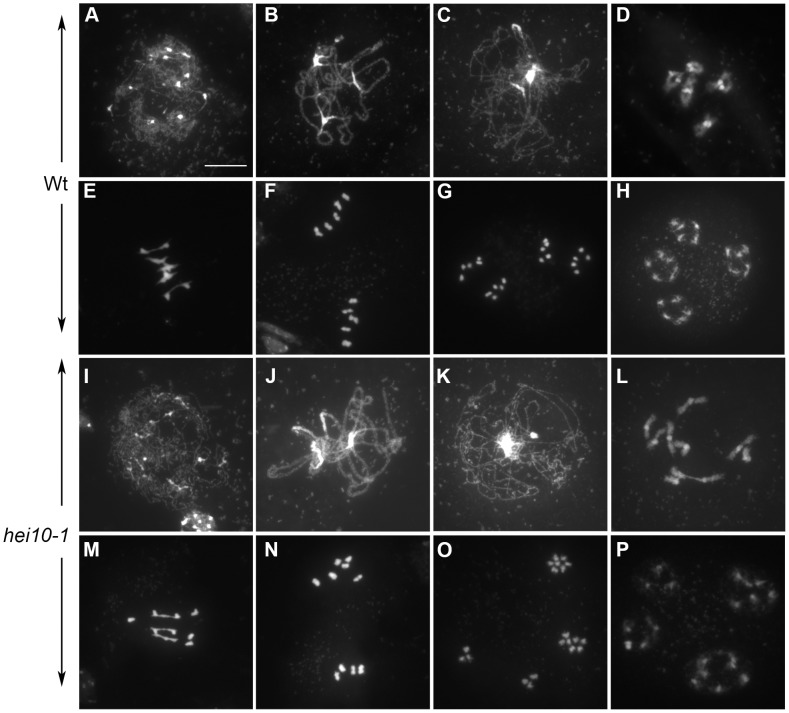
*hei10* mutants show a reduction in chiasma formation. DAPI Staining of wild-type (Wt, Ws-4, A-H) and *hei10-1* (I, P) PMCs during meiosis. A, I: leptotene; B, J: Pachytene; C, K: Diplotene; D, L: Diakinesis; E, M: Metaphase I; F, N: Metaphase II; G,O: Anaphase II; H, P: Telophase II. Heterochromatin (rDNA and centromeres) is stained more brightly than euchromatic arm regions. Bar: 10 µm.

Next, we investigated male meiosis by staining chromosomes with 4′,6-diamidino-2-phenylindole (DAPI). Wild-type *Arabidopsis* meiosis was described in detail in [40], and the major stages are summarized in Figure 2A–2H). During prophase I, meiotic chromosomes condense, recombine, and undergo synapsis, resulting in the formation of five bivalents, each consisting of two homologous chromosomes attached to each other by sister chromatid cohesion and chiasmata, which become visible at diakinesis (Figure 2D). Synapsis (the close association of two chromosomes *via* an SC) begins at zygotene and is complete by pachytene (Figure 2B), by which point the SC has polymerized along the whole length of the bivalents. At metaphase I, the five bivalents are easily distinguishable (Figure 2E). During anaphase I, each chromosome separates from its homologue, leading to the formation of dyads corresponding to two pools of five chromosomes. The second meiotic division then separates the sister chromatids, generating four pools of five chromosomes (Figure 2F and 2G), which gives rise to tetrads of four microspores (Figure 2H).

In *hei10* mutants, the early stages of meiosis could not be distinguished from wild type: chromosomes appeared as threads at leptotene (shown for *hei10-1* on Figure 2I), that condensed and synapsed until pachytene (Figure 2J). Aberrations, however, appeared at early diakinesis in *hei10* mutants, with cells showing a mixture of bivalents and univalents (Figure 2L). At metaphase I, this defect became even more obvious with mutant cells showing a variable number of univalents (Figure 2M). Subsequently at anaphase I, the univalents segregated randomly in the mutant (Figure 2N). Then, in the second meiotic division, sister chromatids segregated normally (Figure 2O), giving rise to daughter cells containing aberrant numbers of chromosomes (compare Figure 2P). Female fertility was also investigated in *hei10* mutants and found to be drastically reduced in all five alleles due to abortion of a large proportion of female gametophytes (not shown).

We estimated the mean number of chiasmata at metaphase I on spread PMC chromosomes counterstained with DAPI as described in [41] in all *hei10* mutants and their respective wild-type accession. As shown in Table 1, we observed a significant decrease in chiasma formation in every mutant in comparison to wild types. In *hei10^Ws^* alleles, we measured a mean of 0.64 chiasma/cell which corresponded to a 12 fold decrease or a 8.5% residual chiasma level in comparison to wild type. For the Col-0 allele, we observed a 6.7 decrease in chiasma formation which corresponded to a level of 15% residual chiasma. Statistical analyses on these data showed that there was no difference among the four *hei10* alleles in the Ws background (Table 1, p = 0.4955).

**Table 1:** Chiasma shortage in *hei10* mutants.

	Ws-4	*hei10-1*	*hei10-3*	*hei10-4*	*hei10-5*	Col-0	*hei10-2*
Chiasma/cell	7.4	0.62	0.68	0.74	0.54	9.2	1.37
SD	1	0.75	0.77	0.83	0.79	1	1.11
n	38	92	54	65	72	55	56

SD: Standard Deviation.

We then measured the level of recombination on two intervals on chromosome 5, using the Fluorescent Tetrad Lines (FTL) which contain multiple fluorescent markers expressed in pollen. Combined with the quartet (*qrt*) mutation which prevents the four pollen grains from separating, these lines allow recombination between the linked fluorescent markers to be scored directly by looking at pollen tetrad fluorescence, thereby facilitating scoring of a large number of meiotic products [42].

The two intervals tested here correspond to intervals I5a and I5b described in [42]. They are located at the bottom of the chromosome 5. Because the FTL lines were developed in a Col-0 background, we used *hei10-2* allele for this quantification. We crossed *hei10^+/−^ qrt1–2^−/−^* to an FTL line with three linked insertions on chromosome 5 (also in the *qrt1–2^−/−^* background, see materials and methods), each encoding a different colour fluorescent protein (RFP, YFP and CFP). The resulting tetrad data was analyzed using the Perkins mapping equation based on the measurements of tetratype, parental, and non parental ditype combinations of markers [43].

As shown in Table 2, *hei10-2* mutants showed a drastic reduction in the genetic distance in both chromosome 5 intervals: a 3 times decrease on I5a and a 2.3 times decrease on I5b which confirms the overall decrease of CO formation previously measured by chiasma counting. The decrease is less drastic than that measured by chiasma counting. However, this can be explained by the difference in methods since chiasma counting considers 100% of the male meiocytes at a given stage of meiosis (metaphase I-anaphase I transition) while tetrad analyses is performed only on viable tetrads.

**Table 2:** Recombination rates in the *hei10* mutant.

		Nb of tetrads	PD	TT	NPD	d (cM)^1^
I5a	wt	11,672	6,746	4,845	81	**22.84**
	*hei10-2*	3,127	2,662	461	4	**7.76**
I5b	*wt*	11,672	8,818	2,825	29	**12.85**
	*hei10-2*	3,127	2,794	331	2	**5.48**

1 calculated using the Perkin equation (Perkins 1949 Genetics 34:607).

PD: Parental Ditype, TT: TetraType, NPD: Non Parental Ditype.

**Figure 3:**
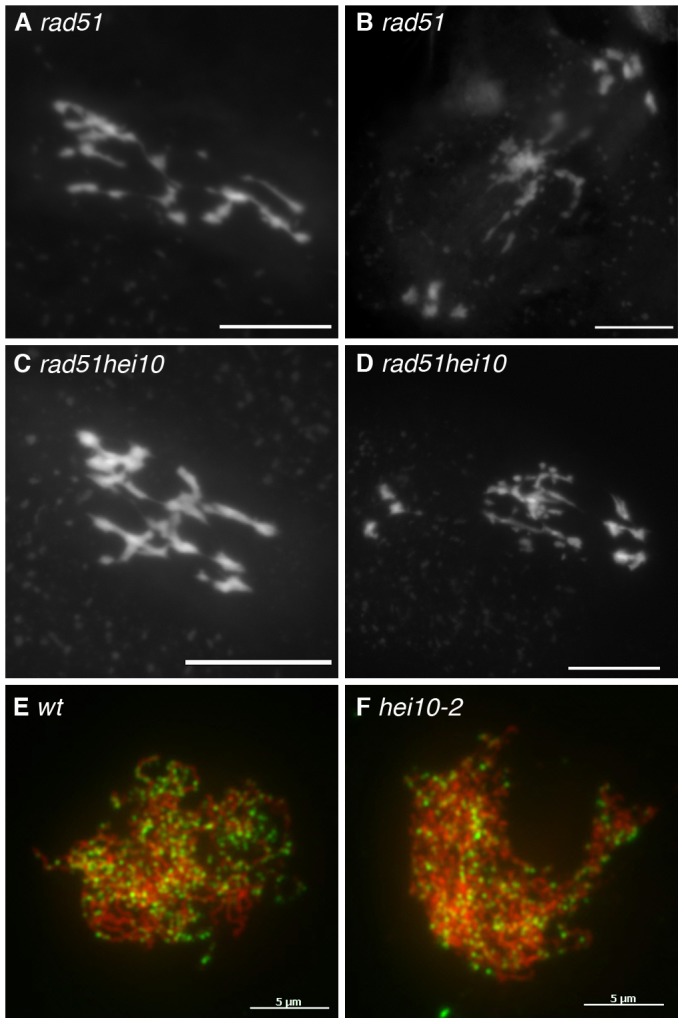
Early recombination defects are not detected in *hei10* mutants. DAPI staining of Metaphase I (A, C) and Anaphase I (B, D) PMCs in *rad51* (A, B) or *rad51hei10* (hei10-2 allele, C, D) mutants. Bar: 10 µm. Co-immunolocalisation of ASY1 (red) and AtDMC1 (green) in wild-type (wt, E) and mutant meiocytes (*hei10-2*, F). Bar: 5 µm.

In order to examine whether this decrease in recombination is due do defective recombination initiation, we investigated DSB formation by introgressing the *hei10-1* mutation into an *Atrad51* mutant, defective for meiotic DSB repair [44]. It was shown previously that in this background, DSBs are formed but are then processed abnormally, leading to significant chromosome bridges and pronounced chromosome fragmentation during anaphase I ([44] and Figure 3A and 3B). This DNA fragmentation persisted in *hei10rad51* (Figure 3C and 3D) demonstrating that DSBs are present in the *hei10* mutant, even if we cannot exclude minor effects on the level of DSB formation. Since this technique is poorly quantitative, we also analysed the nuclear distribution of the DMC1 protein, which is an essential component of the recombination machinery. Its appearance on meiotic chromosomes during prophase is thought to mark the sites of recombination repair. To follow DMC1 focus formation throughout meiosis, co-immunolocalisation was performed with an antibody that recognises the meiotic protein ASY1, a protein associated with the axial element of the SC [45]. Detailed analysis of DMC1 progression in wild-type *Arabidopsis* meiotic prophase was described in [32]. DMC1 foci appear at late leptotene/early zygotene reaching an average of 240 foci per nucleus and disappear by pachytene. In *hei10* mutants, the DMC1 foci appeared at the same stage as in wild type (Figure 3E and 3F) and their average number was 257+/−51 (n = 13), which was not statistically different from wild type (t test, p = 0.43352).

From these data, we can conclude that in *hei10* mutants 85 to 90% of the COs are absent, and that this recombination defect is not correlated with major defects in the initiation of recombination events.

### HEI10 is necessary for Class I COs

In *Arabidopsis*, most *zmm* mutations studied so far suppress 85–90% of the COs (depending on the genetic background Col-0 versus Ws-4), except *mer3* which suppresses 71–76% of COs ([30] and Table 3). In order to understand HEI10 function during meiotic recombination, we measured the chiasma level in double *hei10msh4* and *hei10mer3* mutants compared to each single mutant (Table 3, *hei10* epistatic group). We found that residual CO levels in *hei10* were not statistically different from *msh4* but the residual CO levels in these two single mutants was statistically different from that of *mer3*. We also observed that the rate of CO formation in *hei10* is the same as the rate in *msh4hei10* and *mer3hei10* (ANOVA on SAS procedure GLM Student-Newman-Keuls grouping). As a control, we verified that the residual COs in *hei10* were abolished in the double *hei10spo11-1* showing that they are dependent on SPO11-1 (Table 3, *hei10* epistatic group).

**Table 3:** HEI10 is a ZMM protein.

*hei10* belongs to the same epistatic group as *zmm* mutants *msh4* and *mer3*										
	Ws-4 ecotype							Col-0 ecotype		
	*hei10-1*	*spo11-1*	*msh4*	*mer3*	*hei10* *spo11-1*	*hei10msh4*	*hei10mer3*	*hei10-2*	*msh4*	*hei10* *msh4*
chiasma/cell	0.62	0.5	0.91	2.12	0	0.7	0.69	1.37	1.29	1.53
SD	0.75	0.7	0.95	1.09	0	0.85	0.89	1.11	0.96	0.89
n	92	30	64	42	89	96	134	56	79	75

Crossovers in *hei10* are less interferent than in wild type			
	Map distance without adjacent CO	Map distance with adjacent CO	Interference ratio^1^
I5a Wt	26.9	10.28	0.3823
I5a *hei10-2*	7.93	6.31	0.7955
I5b Wt	17.88	5.96	0.3333
I5b *hei10-2*	5.65	4.52	0.8

SD: Standard Deviation.

1 ratio between map distance with and without adj CO.

We also showed that the level of residual chiasma in *hei10-2^Col0^* was not statistically different to that in *msh4^Col0^* (Table 3, *hei10* epistatic group). Therefore, we can conclude that HEI10 belongs to the same pathway as *MSH4* and *MER3*, and is necessary for the same proportion of COs as *MSH4*.

The observation that CO levels are reduced in *hei10* plants and that *HEI10* belongs to the same epistasis group as *MER3* and *MSH4* would predict that interference between remaining COs is reduced. To test this prediction, we measured the genetic distances of an interval (e.g. I5a) with and without the presence of a simultaneous event in the adjacent interval (e.g. I5b), as described in [42] (Table 2 and Table 3, crossovers). The ratio of genetic distance with the presence of an adjacent CO relative to the distance when an adjacent CO is absent gives the interference ratio. When COs in the two intervals are independent, the interference ratio is 1, whereas the stronger the interference between two adjacent COs, the lower the interference ratio.

In wild type (Table 3, crossovers), chromosomes that recombined in one interval showed a strong decrease in the frequency of COs in the adjacent interval (10.28% *versus* 26.9% for recombination in I5a or 5.96% *versus* 17.88% when analysing recombination in I5b). The wild-type interference ratio was 0.38 for I5a and 0.33 for I5b. The same analyses in *hei10* (Table 3, crossovers) showed that the effect of a CO in an adjacent interval is much lower than in wild type with interference ratios of 0.8 for both intervals (not statistically different from 1, p>0.2 for both intervals). Therefore, HEI10 in *Arabidopsis* is necessary for the normal level of interference.

**Figure 4:**
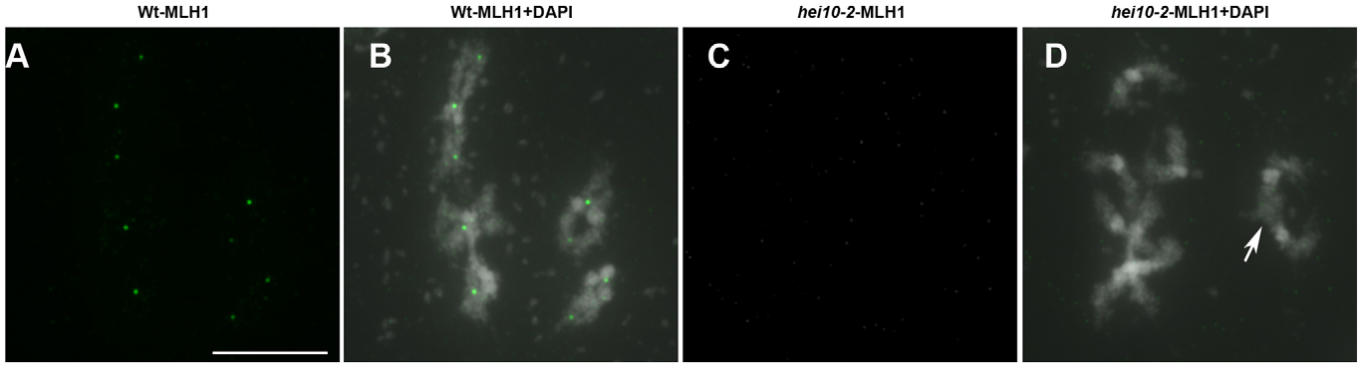
Class I crossovers are absent in *hei10* mutants. Immunolocalisation of MLH1 in wild type (Wt, A–B) and *hei10-2* (C–D) mutant PMCs at diakinesis. For each genotype the MLH1 signal alone (A, C) or a merge signal with DAPI staining is shown. With DAPI, centromeres are stained more intensively than the chromosome arms. The arrow indicates a residual chiasma in *hei10-2*. Bar: 10 mm.

Finally, in order to confirm that HEI10 is necessary for Class I COs, we immuno-labelled *hei10* chromosomes with antibody against MLH1. In meiosis, MLH1 forms foci on chromosomes during prophase, and these foci mark the sites of class I COs in many species including mouse, tomato, and *Arabidopsis* [14], [46], [47]. In *Arabidopsis* MLH1 can be visualised as foci from late pachytene to diakinesis where it co-localises with class I chiasmata ([47], and Figure 4A and 4B). In the *hei10* mutant (Figure 4C and 4D), we never observed a MLH1 signal during *hei10* diakinesis (n = 60 for *hei10-1* and n = 30 for *hei10-2*) confirming that the residual chiasmata observed in these genotypes are likely to be class I-independent.

### The HEI10 protein forms foci on meiotic chromosomes from leptotene to diakinesis

**Figure 5:**
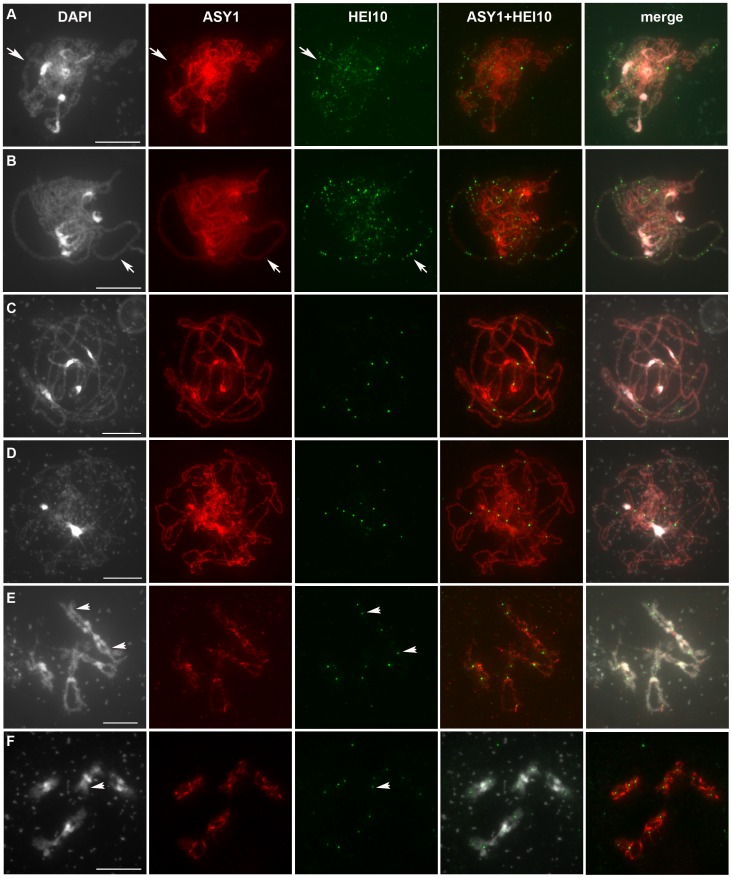
HEI10 can be detected on chromosomes throughout the entire meiotic prophase. Co-immunolocalisation of ASY1 and HEI10 on wild-type Col-0 PMC chromosomes after acetic acid spreading. For each cell: DAPI staining, ASY1 labelling, HEI10 labelling, merge ASY1 and HEI10 (ASY1+HEI10) or the three merged signals are shown. With DAPI, heterochromatin (centromeres and repeated rDNA regions) is stained more brightly than euchromatin. A: Early Zygotene, B: Late Zygotene, C: Pachytene, D: Diplotene, E–F: Diakinesis. Arrows in A and B indicate synapsed regions, while arrows in E and F indicate HEI10-labelled chiasmata. With DAPI staining, some remaining cytoplasmic components (mitochondria) can appear as bright spots (C–F)). Bar: 10 µm.

In order to analyse HEI10 protein dynamics throughout meiosis, we produced antibodies raised against the full-length *Arabidopsis* protein (see Materials and Methods). The antibodies were affinity purified and used on meiocyte spreads using either lipsol ([45], Figure S7 and S8) or acetic acid spreads ([47], Figure 5). We analysed HEI10 distribution in Columbia (Col-0) and Wassilevskija (Ws-4) ecotypes together with either ASY1 or ZYP1 (the *Arabidopsis* SC central element protein, [48]) for staging.

**Figure 6:**
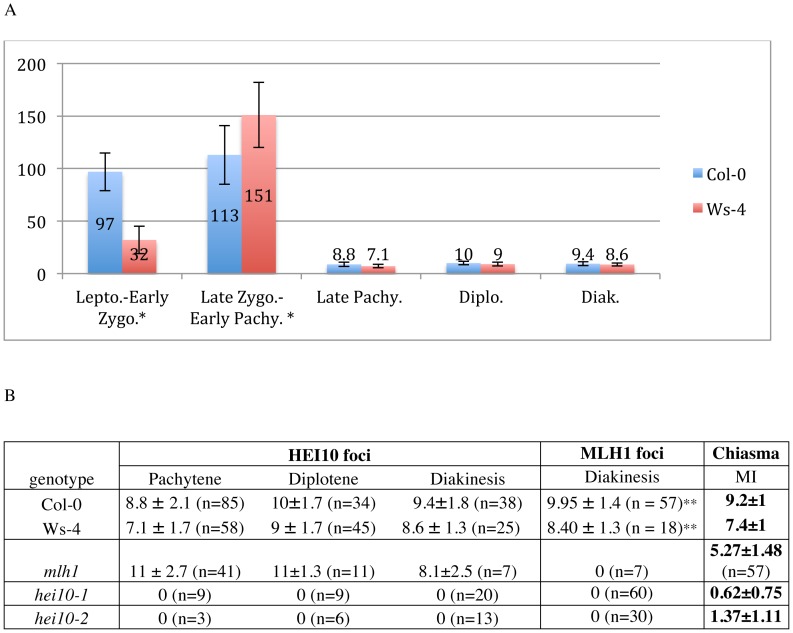
Quantification of HEI10 foci during meiotic prophase. A: HEI10 foci counting at different stage of prophase I. B: HEI10 foci formation during late prophase in various genetic backgrounds. Foci number quantification was undertake on acetic acid spreads except when notified by *. ** According to [47]. MI: Metaphase I.

In Col-0, the HEI10 signal was first detected at stages when ASY1 forms continuous threads corresponding to full axial elements, prior to initiation of synapsis (leptotene, Figure S7A). At this stage, HEI10 appeared as numerous foci on lipsol spreads but not on acetic acid spreads (not shown). Progression into the next step of meiosis was further monitored using anti-ASY1, whose signal becomes fainter while homologous chromosomes synapse (Figure 5A–5B, and Figure S7B, arrows), or with ZYP1 antibodies that mark the central element of the SC (Figure S8). During synapsis initiation, bigger and brighter HEI10 foci were visualised, often co-localising with synapsed regions of the chromosomes (Figure 5A, and Figure S7B). Quantification of HEI10 foci showed that their total number did not vary significantly during early prophase with an average number of 97±18 (n = 64) in Col-0. In Ws-4, HEI10 foci appeared concomitantly with ZYP1 staining (Figure S8) and in smaller numbers than in Col-0 (32±13, n = 37 Figure 6). Then, from mid-zygotene to early pachytene (when synapsis has been achieved), a combination of large and small HEI10 foci were still observed (Figure 5B and Figure S7C–S7D). The average foci number in Col-0 increased slightly during these stages (113±28 n = 41, and was significantly different from foci counts at early zygotene p = 0.002) while in Ws-4, the increase was much more significant (151±31 n = 35, p = 1.5 10^−24^) (Figure 6).

During pachytene stage, the number of HEI10 foci dropped dramatically, and only a few large and bright foci were retained on late pachytene cells (Figure 5C and Figure 6, Figure S7E). This change probably occurred very quickly since no progressive decrease in HEI10 foci could be observed. The mean HEI10 foci number per cell at this stage was 8.8±2.1 (n = 85) for Col-0 and 7.1±1.7 (n = 58) for Ws-4 (counted on acetic acetic spreads) (Figure 6). In some cases, the selection of a few bright foci was observed together with a faint HEI10 signal marking the central element in a near linear manner (Figure S7E).

At diplotene and diakinesis, HEI10 foci were maintained on chromosomes. Diplotene foci are the easiest to score because they are the most homogeneous in size and intensity (Figure 5D and Figure S7F). At diakinesis, chromosome condensation allows the five *Arabidopsis* bivalents to be identified (Figure 5E–5F). At this stage, the mean HEI10 foci number per cell was 9.4±1.8 (n = 38) for Col-0 and 8.6±1.3 (n = 25) for Ws-4 (Figure 6A and 6B, not statistically different from number at diplotene: p = 0.07 for Col-0 and p = 0.15 for Ws-4). At this stage, HEI10 foci appeared to co-localise with sites of homologous connections corresponding to chiasmata (arrow heads on 5E and 5F). Then, at later stages (from metaphase I to the end of meiosis) the HEI10 signal was no longer detected (not shown).

The specificity of the observed signal was confirmed using the *hei10* mutants as negative controls. No signal was ever observed in any of the Ws-4 mutants at any stage (shown for *hei10-1*, Figure S9A–S9E and Figure S10A–S10C). In the case of the *hei10-2* mutant however, a modified signal could still be observed. On acetic acid spreads it appeared as a spotty signal composed of dispersed and small foci surrounding chromosomes or localised on centromeric regions (Figure S9F–S9J). On lipsol spreads it occasionally appeared as a bright signal associated with the central element of the SC at zygotene and pachytene (Figure S10D–S10F). However, we never observed HEI10 foci similar to those observed in wild type. Therefore in the *hei10-2* allele, a modified HEI10 protein is likely to be produced (confirming the RT-PCR data) that is able to load onto the central element of the SC. Nevertheless, this mutated HEI10 appears to be non-functional since the strength of the *hei10-2* mutation is the same as the *msh4* mutation in terms of residual chiasma (see above).

### HEI10 foci colocalise with MLH1 foci from pachytene to diakinesis

**Figure 7:**
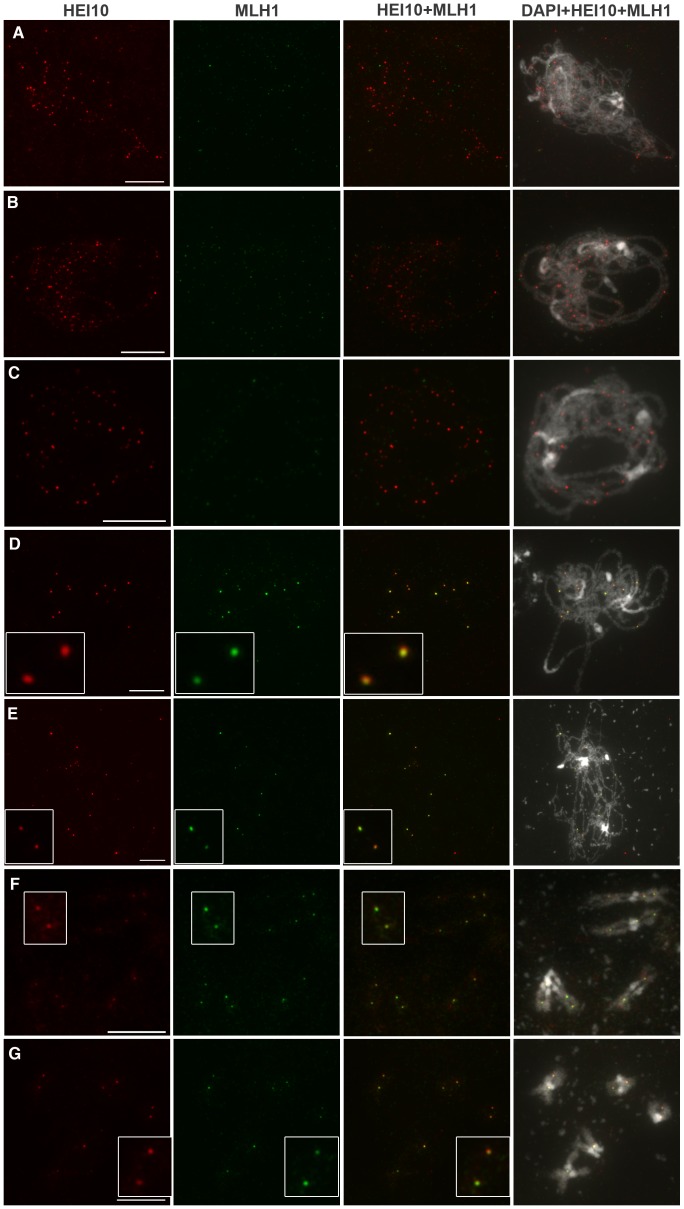
HEI10 and MLH1 co-localise from late pachytene to diakinesis. Co-immunolocalisation of HEI10 (red) and MLH1 (green) on wild-type (Col-0) PMC chromosomes after acetic acid spreading. Bar: 10 µm. For each cell, individual signals are shown as well as the overlay of both signals (HEI10 and MLH1) and merged signals of both antibodies together with DAPI. A:Zygotene, B–C: early Pachytene, D: Late pachytene, E: Diplotene, F–G: Diakinesis. With DAPI, heterochromatin (centromeres and repeated rDNA regions) is stained more brightly than euchromatin. Bar: 10 µm.

From late pachytene to diakinesis, HEI10 forms approximately 10 foci on chromosomes, which is very close in number to our previous reports for the MLH1 protein. Indeed, we recently showed that in *Arabidopsis* MLH1 foci appear at pachytene and reach a maximum at diakinesis, where they mark the site of Class I COs [47]. In order to see if these two proteins label the same sites, we co-immuno-labelled wild-type PMCs with both antibodies (Figure 7). We confirmed that MLH1 appears on chromosomes later than HEI10 since meiocytes from leptotene, zygotene or early pachytene were only labelled with anti-HEI10 and not anti-MLH1 (Figure 7A–7C). The MLH1 signal could be detected on pachytene meiocytes where the number of HEI10 foci had dropped to its “late” amount (probably corresponding to late pachytene) (Figure 7D). From this stage to the end of prophase both proteins were found to co-localise in 100% of cases (Figure 7D–7G).

Taken together, all our findings show that during wild-type meiosis, HEI10 forms foci on meiocytes early during prophase. While recombination progresses, HEI10 staining is retained only at sites that correspond to class I CO sites.

According to the above data, HEI10 and MLH1 both mark class I COs in wild type, but MLH1 appears after HEI10 and at a limited number of sites, whereas HEI10 is loaded early and apparently at numerous sites. This suggests that MLH1 is acting downstream of HEI10. In order to confirm this hypothesis we characterised a mutant allele of *mlh1* (NASC stock centre line SK25975, see Materials and Methods) and showed that disruption of *MLH1* decreases the level of CO formation but to a lesser extent than *hei10* (Figure 6A). Furthermore, MLH1 foci were found to be dependant upon HEI10 while the reverse was not true since HEI10 foci were formed normally in the *mlh1* mutant, showing that MLH1 is indeed acting downstream of HEI10.

### HEI10 belongs to a RING finger protein family largely conserved across kingdoms and related to the Zip3/RNF212 protein family

**Figure 8:**
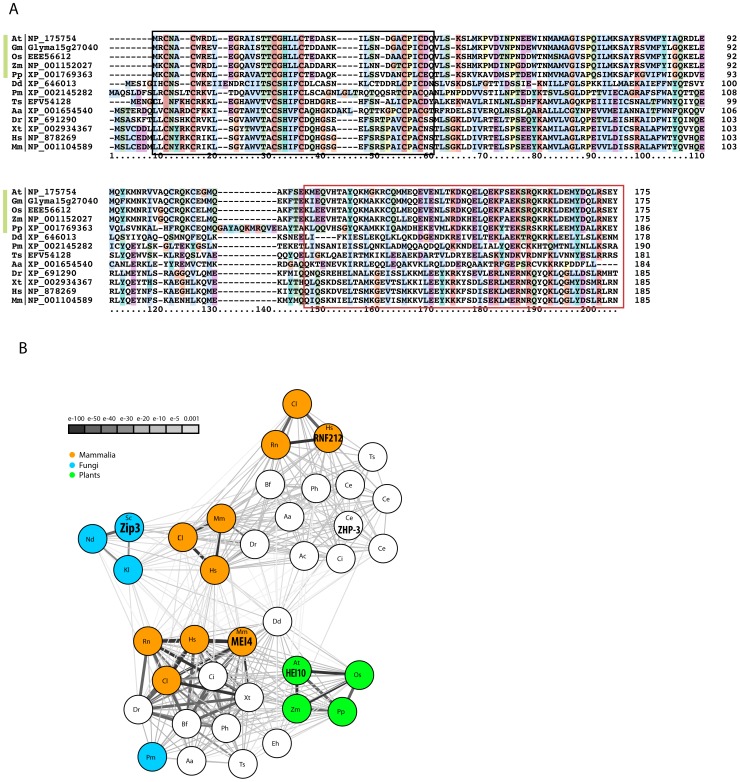
The HEI10 protein family. A: Multiple sequence alignment of HEI10 proteins from plants (green bar, At- *Arabidopsis thaliana*, Gm- *Glycine max*, Os- *Oryza sativa*, Pp- *Physcomitrella patens*, Zm- *Zea mays*), amoeba (Dd- *Dictyostelium discoideum*), fungi (Pm- *Penicillium marneffei*), nematodes (Ts- *Trichinella spiralis*), insects (Aa- *Aedes aegypti*) and vertebrates (Dr- *Danio rerio*, Hs- *Homo sapiens*, Mm- *Mus musculus*, Xt- *Xenopus tropicalis*) showing the conserved N-terminal region including the RING finger (boxed in black) and the coiled-coil forming segment (boxed in red). Protein sequences are listed with a 2-letter species code followed by the reference to the sequence source, numbers indicate amino acid positions. The alignment was generated using muscle and visualized with Clustal [87]. B: BLAST similarity network of HEI10 and Zip3 proteins. The BLAST relationship of all Zip3 and HEI10 homologous proteins from a selected set of species is shown. The nodes represent all Zip3-like (upper panel) and HEI10-like (lower panel) sequences for the predetermined set of species. The edges illustrate their BLAST similarity, where increasing edge width and darker edge colour indicates higher similarity (lower BLAST E-value). The highlighted nodes show the following sequences: mammalian (*Cl*- *Canis lupus*: XP_539671 HEI10, XP_855088 RNF212, XP_549701 C14orf164, *Hs*-*Homo sapiens*: NP_878269 HEI10, NP_919420 RNF212, XP_003119614 C14orf164, *Mm-Mus muscuslus*: NP_001104589 HEI10, D3Z423 C14orf64, *Rn- Rattus norvegicus*: XP_002728107 Rnf212), fungi (*Nd- Naumovozyma dairenensis*: CCD27121, *Kl*- *Kluyveromyces lactis*: XP_451682, *Sc- Saccharomyces cerevisiae*: NP_013498 Cst9 = Zip3, *Pm*- *Penicillium marneffei*: XP_002145282), plants (*At*- *Arabidopsis thaliana*: NP_175754 AT1G53490, *Os- Oryza sativa*: NP_001046371, *Pp- Physcomitrella patens*: XP_001769363, *Zm- Zea mays*: NP_001152027) and nematode (*Ce- Caenorhabditis elegans*: NP_492311 ZHP-3). Among these, ScZip3 [24], CeZHP-3 [88], MmHEI10 (MEI4, [39]), AtHEI10 (this study) and HsRNF212 [71] have been shown to be involved in meiotic recombination. Other species code: Aa- *Aedes aegypti*, Ac- *Angiostrongylus cantonensis*, Bf- *Branchiostoma floridae*, Ce- *Caenorhabditis elegans*, Ci- *Ciona intestinalis*, Dr- *Danio rerio*, Dd- *Dictyostelium discoideum*, Eh- *Entamoeba histolytica*, Ph- *Pediculus humanus*, Ts- *Trichinella spiralis*, Xt- *Xenopus tropicalis*.

The cDNA clone BX814259 corresponding to the full length At1g53490 gene was obtained from the CNRGV (http://www.genoscope.cns.fr/cgi-bin/ggb/arabidopsis/gbrowse/arabidopsis/) and fully sequenced, confirming that At1g53490 encodes a protein of 304 amino acids (Accession number NP_175754). Similarity to a RING finger domain is predicted in the N-terminal region of At1g53490 using Pfam domain analysis (Pfam 26.0, PF13923/zf-C3HC4_2/RING finger, pos 1–43, E = 2.1e-07) (Figure 8A) [49]. Using Marcoil with default parameters and a 90% stringency level, NP_175754 is predicted to contain a coiled-coil forming domain from 117 to 177 (Figure 8A) [50].

Likely orthologues of At1g53490 can be identified in major eukaryotic kingdoms- amoeba, plants, animals and fungi- using either automated orthology prediction tools (e.g. ORTHOMCL v5 and eggNOG 3.0) or via a Reciprocal Best BLAST approach [51]. The human orthologue of At1g53490 is known as CCNB1IP1 (as well as HEI10 (for Enhancer of cell Invasion N°10). All the At1g53490 orthologues obtained share a highly conserved N-terminal region, as can be seen in the multiple sequence alignment in Figure 8A. A more precise investigation of the occurrence of At1g53490 in various *phyla*, identified similar sequences in very distant ones including Ascomycetes, Arthropods and Nematodes. However we could not identify At1g53490 orthologues in model organisms of the same *phylum* as *Saccharomyces cerevisiae*, *Schizosaccharomyces pombe*, *Drosophila melanogaster* or *Caenorhabditis elegans* was unsuccessful, suggesting that At1g53490 was lost by these species.

In order to identify more distant sequence homologues of HEI10 proteins, the alignment of the conserved HEI10 N-terminal region as shown in Figure 8A was used to start single-round PSI-BLAST searches on various species-specific protein sets [52]. A PSI-BLAST search of the human subset of NCBI-nr, in addition to hitting HEI10/CCNB1IP1, yields two other significant hits with E<0.001, C14orf164-like LOC100507650 (E = 4e-08, XP_003119614) and C14orf164 (E = 1e-07, A8MTL3). XP_003119614 and A8MTL3 map to the same gene in the human genome. A PSI-BLAST search of the *S. cerevisiae* proteome (SGD) obtains three significant hits (E<0.001): Cst9p/ScZip3 (E = 3e-06, Accession N° NP_013498, alignment length 142 aa), RAD18 (E = 4e-05, Accession N° CAA31059 alignment length 52 aa), and BRE1 (E = 7e-04, Accession N° Q07457, alignment length 54 aa), but the sequence similarity extends beyond the RING finger domain only in the case of Cst9/Zip3 (not shown). A PSI-BLAST search of the *C. elegans* subset of nr identifies the putative Cst9 orthologue ZHP-3 (E = 5e-05, NP_492311) as the most significant protein hit, in addition to its wormbase-reported ZHP-3 paralogues (F55A12.10, Y39B6A.1, D1081.9) with borderline or below significance similarities. Cst9 *in S. cerevisiae*, ZHP-3, Y39B6A.16, F55A12.10, D1081.9 in C. *elegans* and the C14orf164, RNF212 genes in *H. sapiens* all belong to the Zip3-like protein family in the Panther 7.0 protein classification (PTHR22663) [53]. Thus, based on these sequence similarities, HEI10 appears to be a member of the Zip3 protein family. The similarity is found to be reciprocal as HMMer2 searches with the PTHR22663 (Zip3-like) model against the human subset of the NCBI nr (non-redundant database) results in significant hits with proteins of the Cst9/ZHP-3 family (genes RNF212 and C14orf164/LOC100507650) and human HEI10 (NP 878269, E = 0.0002). In the *Arabidopsis* proteome the same model obtains only one hit below the significance threshold, At1g53490 (E = 0.00018). The sequence relationship of the two groups of proteins, which share a N-terminal RING domain followed by a conserved helix-containing segment, a predicted coiled-coil forming region and a variable C-terminal stretch, is further illustrated in Figure 8B.

## Discussion

### Identification of a new conserved ZMM protein

We have shown that, in *Arabidopsis*, HEI10 is necessary for 85–90% of meiotic COs (depending on the genetic background), while early events in meiotic recombination do not appear to be affected. Therefore it appears that in the *hei10* mutant backgrounds, DSBs are formed normally and efficiently repaired (since no fragmentation is ever observed) but the CO output is drastically limited. We can speculate that the few breaks normally channelled to COs will either yield NCOs or be repaired on the sister chromatids. We also showed that in the *hei10* mutant, residual COs measured on two adjacent intervals were largely interference-insensitive. Additionally, we showed that the remaining chiasmata in *hei10* mutants were not labelled by anti-MLH1 antibodies. Lastly, we demonstrated that *hei10* belongs to the same epistatic group as two other *zmm* mutants, *msh*4 and *mer3*, and that *hei10* is epistatic to *mer3* in terms of residual CO levels. The *hei10* mutant phenotype is therefore indistinguishable from those of *msh4*, *msh5*, *zip4*, *shoc1* and *ptd* in terms of CO outcome and an absence of synapsis defects [10], [11], [32], [35], [36], [54], showing that *hei10* possesses all the characteristics of an *Arabidopsis zmm* mutant.

Proteins with strong similarity to HEI10 could be identified in numerous species, from fungi to vertebrates. They are small coiled-coil proteins characterised by an N-terminal RING finger domain. Functional data on members of this family are scarce, but several data point to a role in cell cycle control in mammals (where it has also been called CCNB1IP1 [55], for Cyclin B1 Interacting Protein 1). Firstly, HEI10 (for Enhancer of cell Invasion N°10) was identified in a screen for human cDNAs promoting yeast agar invasion and filamentation [56]. *HEI10* was also found to be overexpressed in melanoma [57], and heterologous expression led to a model in which HEI10 regulates G2/M transition in mammals by controlling Cyclin B1 levels [55], [56], [57]. Sing et al. [55] also showed that HEI10 is required for cellular proliferation, and that unlike in yeast it is a negative regulator of invasion in mammalian cells. In the case of *Arabidopsis* HEI10, we did not obtain evidence that this protein plays a somatic role. Nevertheless, further studies are needed to definitely rule out any involvement of HEI10 in the plant somatic cell cycle. In addition to its possible involvement in cell cycle control of mammalian cells, HEI10 was also shown to be necessary for reproductive development in mammals since a point mutation in the mouse *HEI10* was shown to be responsible for the meiotic defect of the *mei4* mutant [39].

The mouse *mei4* mutant shows normal synapsis but a strong decrease in bivalents at diakinesis during male meiosis. RAD51 foci form normally but there are no MLH1 or MLH3 foci formed at pachytene. Lastly, CDK2 labelling is abnormal and spermatocytes arrest at metaphase I [39]. From these results, Ward and collaborators proposed that HEI10 functions to couple MMR to the cell cycle machinery, targeting any of the numerous mammalian cyclin-B related proteins for degradation. In light of our data, obtained on HEI10 function during *Arabidopsis* meiosis, it is more likely that HEI10 is also a component of the class I CO pathway in mammals. Since *mei4* meiosis defects are indistinguishable from those in *mlh1* or *mlh3* knockouts [17], [21], [58] but different from *msh4* and *msh5* (which display synapsis defects and meiotic arrest at zygotene [12], [28], [59], [60]), it could be suggested that HEI10 in mammals is necessary along with MLH1 and MLH3 for late steps of CO maturation. This function would also be compatible with its expression in testis from 15 dpp (days post partum) corresponding to meiocytes entering pachynema [61].

### HEI10 is a class I CO marker, loaded early during recombination

In most species the number of recombination sites during meiosis is in large excess compared to the final CO number [62], [63]. The study of the dynamics of recombination proteins during meiosis showed that recombination is initiated at a large number of DSBs which are then processed, generating free 3′DNA ends rapidly coated with recombinases (RAD51 and DMC1, corresponding to the approximately 300 ENs in mouse [64], or the 250 DMC1 foci in *Arabidopsis* [32]). Later on, the decrease in RAD51/DMC1 foci from leptotene to pachytene is thought to reflect the progression of the repair events. The appearance of MSH4 foci (between 100 to 150 in mouse as in *A. thaliana*) is then likely to mark the intermediate stages of recombination involving strand exchange, while CO maturation is thought to happen in MLH1-containing late recombination nodules (LNs) [15], [46]. We showed that HEI10 is a ZMM protein which appears as numerous foci (113±28 in Col-0, and 151±31 in Ws-4) on male meiocytes during leptotene and zygotene, as is the case for AtMSH4 [10]. Then, when synapsis is complete, the number of HEI10 foci decreases dramatically so that foci are seen persisting at only approximately 10 sites, and unlike MSH4, all these sites co-localise with MLH1. The perfect co-localisation of HEI10 and MLH1 is observed until diakinesis. Thus, HEI10 appears unique among the ZMM described to date: it is not only loaded early during recombination but also persists until the very end of recombination.

From a genetic point of view, *hei10* mutants behave like most of the other *Arabidopsis zmm* mutants described so far: *msh4*, *msh5*, *shoc1*, *ptd* and *zip4* [10], [11], [32], [35], [36], [54]. This suggests that they are all likely to be involved at the same step of meiotic recombination, that is the choice and the maturation of recombination intermediates of the class I pathway, which represent 85% of the total amount of COs in *Arabidopsis*. Four other proteins are involved in CO I formation in *Arabidopsis*, but to a lesser extent: MER3, RPA1, MLH1 and MLH3, which are necessary for 75 to 40% of the total amount of the COs ([19], [29], [30], [65] and this study). Our knowledge of the biochemical functions of these proteins in budding yeast lead to a model in which the Mer3 helicase would act early after the D loop formation to stimulate the heteroduplex extension, stabilising nascent D loop structures [33]. Msh4 and Msh5 are known to form a heterodimer that could act as a clamp to hold homologous chromosomes together, thereby stabilising the Holliday junction and facilitating CO formation [34]. Lastly, the XPF endonuclease related Zip2/SHOC1 was proposed to stabilise double Holliday junctions *via* its involvement with the ERCC1-like protein PTD [35], [36]. Therefore, the current model is that ZMM proteins act at CO-designated sites to stabilise recombination intermediates channelling them irreversibly towards CO maturation.

HEI10 is therefore very likely to play a role at this step of recombination. Nevertheless, as already mentioned, HEI10 protein dynamics are very different from those of other ZMMs. In *S. cerevisiae*, ZMM proteins form a limited number of foci which are very likely to represent class I COs [9]. In mammals and plants, the situation however is very different since MSH4/5 proteins are loaded onto a much larger number of recombination sites than the final number of class I COs. Furthermore, in mammals, MSH4 foci were shown to display some interference [14], while MLH1 showed even stronger interference. This suggests that, in these species, approximately half of the initial DSB sites are selected and establish a first layer of interference. Then, out of these, only a few will mature into strongly interfering class I COs, while the remaining will undergo NCO repair. This model probably also applies to plant intermediates of recombination, as suggested by the high number of HEI10, MSH4 and MSH5 foci formed at early prophase (here and [10]). The situation in *Sordaria* where MER3 and RAD51 foci were recently shown to co-localise in more than 90% of the cases, led to the hypothesis that after the establishment of nascent interactions between homologous chromosomes by the RECA homologues, MER3 and then MSH4/5 proteins would act to place the resulting structures in an appropriate conformation for the subsequent recombination events. Assuming that early staining with HEI10 represents early selection of recombination intermediates to be channelled into the ZMM pathway, HEI10 would therefore generate continuity between chosen early recombination intermediates and class I COs, making it a backbone component of the ZMM pathway.

This early loading of HEI10 is however difficult to reconcile with the late phenotype of *hei10* mutants. Indeed, no early defects could be detected in our study of prophase in the *hei10* mutant. This could mean that HEI10 is recruited at multiple DSB sites to govern the choice of repair outcome, i.e. CO versus NCO. This would then suggest that the fate of recombination sites that will yield class I COs is determined very early after DSB formation. Alternatively we cannot exclude that HEI10 also plays a role during these earlier stages of prophase as was shown recently for three ZMM proteins in *Sordaria* (MER3, MSH4 and MLH1) in [66]. This study revealed unsuspected early roles in early homology pairing (MER3 and MSH4) or interlock maturation (MLH1) in addition to their already known role in CO maturation at later steps of prophase progression [66].

### Is HEI10 a functional homologue of Zip3?

The bioinformatics studies presented in this paper showed that the Zip3 and HEI10 protein families can group together. This can be explained by overall similarities in their protein sequences with an N-terminal RING finger domain, and a central coiled-coil region [37], [56]. Nevertheless, the Zip3 RING domain was associated with a SUMO E3 ligase activity [38], while HEI10 was shown to possess a ubiquitin ligase activity *in vitro* [56], even if a possible SUMO E3 ligase function was also recently suggested in mammals [61].

Besides these structural similarities, there is a clear convergence between the functions of these two classes of proteins. First, Zip3 in *S. cerevisiae*, Zhp-3 in *C. elegans*, and HEI10 in mouse and *Arabidopsis* are all required for wild-type CO levels. Furthermore, in yeast, nematodes and plants, they are exclusively necessary for the formation of interfering COs. Even more striking, in these latter species Zip3/ZHP-3 and HEI10 were shown to be very good markers of these class I COs as soon as late pachytene and until diplotene or diakinesis depending on the species considered ([24], [67] and this study). Lastly, studies in yeast proposed that Zip3 acts upstream of other Zmms [24], [68], which is also likely to be the case for HEI10 according to our immunolocalisation results in *Arabidopsis*.

Divergences nevertheless appear when one considers the involvement of these proteins in SC formation. In *S. cerevisiae* synapsis initiation takes place from recombination intermediates also destined to be future class I COs (the SICs) [9]. In plants, however, CO formation and synapsis can be uncoupled even if recombination is necessary for synapsis, and it is likely that synapsis proceeds from earlier intermediates [11], [32], [69]. In *C. elegans* where synapsis is independent of recombination [70], Zhp-3 could play a role in coupling CO formation with SC disassembly [67] showing that discrepancies not only occur among the two RING finger protein families but also within the Zip3 family itself.

From all the data available at present, it therefore seems likely that plants, on one hand, and budding yeast and nematodes, on the other hand, have retained two different RING finger proteins to promote essentially the same step of class I CO formation. The identification of the sumoylated/ubiquitinated target(s) of these proteins would definitively confirm this hypothesis. Interestingly, if our assumption that HEI10 and Zip3 are functional homologues proves correct then the situation in mammals is striking since mammals (and other vertebrates) representatives of both families are found in their genome. We have already discussed (see above) the role of mammalian HEI10 during mammalian meiotic recombination, but a possible role for the Zip3 homologue (RNF212) has also been reported in humans, where variants in its sequences were correlated with genome-wide recombination variations [71].

### Original recombination dynamics in plants?

Recombination nodules (RNs) are proteinaceous structures which are visibly associated with the SC and are very likely to be the sites where meiotic recombination occurs [72], [73]. Several classes of RNs can be distinguished (early (EN) and late (LN)) according to the timing of their appearance, number, size, shape, components and their association with either unsynapsed and/or synapsed chromosomal segments [73], [74].

Late Nodules (LNs) of recombination appear at pachytene and remain until early diplotene [74]. These are thought to arise from a fraction of ENs and thought to correspond to CO formation sites. Immunofluorescence studies showed that the MLH1 protein usually follows the same dynamics as LNs. For example, during male meiosis in mouse, MLH1 foci are detected on spermatocytes from mid to late pachytene [17]. The available molecular data from *S. cerevisiae* and mammalian male meiosis also agrees with the cytological dynamics of recombination concerning the timing of DNA repair events. In these two cases, mature COs were detected as soon as pachytene [75], [76].

Nevertheless, in a number of species the dynamics of LN appearance may differ. For example, in female mouse meiosis, MLH1 foci appear earlier at mid-zygotene and persist longer until diplotene where they co-localise with chiasmata visible at this stage in mouse [17], [77], when mammalian oocyte development is blocked until puberty. This was also the case in *Sordaria macrospora* [77] or *Neurospora crassa* [78] where LNs were shown to persist until diplotene. In *Arabidopsis*, we previously reported that MLH1 foci are detected on male meiocyte spreads from pachytene to the end of prophase where 100% of the diakinesis chiasmata are labelled by MLH1 [47]. Similarly here, in this study we showed that HEI10 foci persist until diakinesis. Furthermore, MLH1 timing is similar in *Brassica napus* [79], suggesting that in these two plant species, LNs during male meiosis are likely to be formed during pachytene and to persist until the end of diakinesis. Assuming that these cytological observations of the dynamics of recombination proteins/structures parallel the molecular progression of recombination events, DNA repair timing may vary considerably not only between species but also between sexes within a same species. Therefore the major modifications to chromosome structure (axis set up, synapsis progression, condensation etc…) and the progression of meiotic recombination (strand invasion, second end capture, Holliday junction formation or not, maturation of the COs) could be uncoupled. Another possibility is that LNs also play a structural role in maintaining homologous associations at chiasmata sites from diplotene to diakinesis, well after recombination has been accomplished.

## Materials and Methods

### Plant material

Lines EQO124 (*hei10-1*), EHH69 (*hei10-3)*, EHH9 (*hei10-4*), EVM265 *(hei10-5)*, EXY25 (*msh4^Ws^*), EGX254 (*mer3^Ws^*), and FLAG 555F02 were obtained from the Versailles collection of *Arabidopsis* T-DNA transformants (Ws-4 accession) available at http://www-ijpb.versailles.inra.fr/en/sgap/equipes/variabilite/crg/ [80].

Lines N514624 (Salk_014624, *hei10-2*) and SAIL_759_A02, SK25975 (seed stock CS1008089, *mlh1-2*) were obtained from the collection of T-DNA mutants from the Salk Institute Genomic Analysis Laboratory (Columbia accession) (SIGnAL, http://signal.salk.edu/cgi-bin/tdnaexpress) [81] and provided by NASC (http://nasc.nott.ac.uk/). The Ws allele of *AtSPO11-1* (*spo11-1-1*) was described in [69]. The *msh4^Col^* mutant corresponds to line Salk_136296 and was described in [10]. The *rad51* mutant was described in [44].

### Growth conditions

*Arabidopsis* plants were cultivated in a greenhouse or growth chamber under the following conditions: photoperiod 16 h/day and 8 h/night; temperature 20°C day and night; humidity 70%.

### *HEI10* cloning

In a screen for *A. thaliana* T-DNA (*Agrobacterium tumefaciens* transferred DNA) insertions that generate meiotic mutants, we isolated four mutant lines (EHH9 = *hei10-1*, EHH69 = *hei10-3*, EQO124 = *hei10-4* and EVM265 = *hei10-5*) that segregated 3∶1 (indicating a single recessive mutation) for reduced fertility and meiotic defects. Linkage analysis between the mutations and the BASTA resistance gene carried by the T-DNA insertions (as described in [69]) showed that none of the mutations were linked with BASTA resistance, suggesting that the T-DNA insertions in these mutants were truncated or aborted.

Rough positional cloning of the *hei10-1*, *hei10-3*, *hei10-4*, *hei10-5* mutations was carried out as described in [82] on mutants selected among an F2 population (*hei10* mutants crossed to wild-type Col-0 accession). The most closely linked marker was chr1_20384267 for all four mutants (based on 30 plants for *hei10-1*, 30 for *hei10-3*, 28 plants for *hei10-4* and 22 plants for *hei10-5*). Fine gene mapping was then carried out using additional semi-sterile plants that were genotyped for microsatellite markers in the selected genomic region. A 400 kb region on chromosome 1 between markers F8L10 (chr1-19,809,520 bp) and F15I1 (chr1-20,209,362 bp) was defined in an F2 mapping population containing 92 mutant plants. According to TAIR10 this region is predicted to contain 124 genes (http://www.arabidopsis.org/). Among these, At1g53490 annotated as a putative DNA-binding protein was selected as the best candidate. Sequencing of At1g53490 in the four mutant lines showed that all four are disrupted in this open reading frame (see below). One other insertion line in At1g53490 available in the public databases (http://signal.salk.edu/), Salk_014624, displayed the same meiotic phenotype as the previously isolated lines, while line SAIL_759_A02 obtained from (http://arabidopsis.info/students/paaras/sail.htm) with a T-DNA insertion in the last intron as well as line FLAG 555F02 obtained from the *Arabidopsis thaliana* Resource Centre for Genomics (http://dbsgap.versailles.inra.fr/portail/) with a T-DNA in the 5′UTR (Figure 1) did not display any fertility defects. Complementation tests (see below) confirmed that the mutations in lines EHH9, EHH69, EQO124 and EVM265 were allelic to Salk_014624.

### Molecular characterisation of the *hei10* alleles

Sequencing of At1g53490 in *hei10-1* mutant line revealed a 44 pb deletion in the third exon of the gene, corresponding to the nucleotides just downstream of the start codon (Figure S1).

The left border of the insertion in the *hei10-2* mutant was sequenced and showed that in this line the T-DNA was inserted in the ninth exon of At1g53490 (Figure 1, nt 1121 of the cDNA). The right border could not be amplified, but PCR with primers P19R and P6 amplified a band of the expected size in the homozygous mutant (data not shown) showing that no major deletions occurred on the other side of the T-DNA.

In *hei10-3* a single nucleotide insertion occurred in the eighth exon of At1g53490 (nt 2626 of the cDNA) leading to a premature stop codon at amino acid 227.

No part of At1g53490 could be amplified for the *hei10-4* and *hei10-5* alleles showing that mutagenesis led to genomic deletions of this region. Using PCR analyses we found that a 8.7 kb deletion occurred (from nt 19,962,396 to nt 19,971,065) removing At1g53490 but also At1g53480, At1g53500 as well as the 5′ end of At1g53510 in *hei10-4* (Figure S3). For *hei10-5*, a larger deletion of more than 13 kb occurred removing At1g53490 and surrounding genes (Figure S4). As expected no *HEI10* transcript is produced in either of these two alleles (Figure S1).

### PCR genotyping of mutant lines

For *hei10-1*, wild-type and mutant alleles were amplified with primers T3F20-P21 and T3F20-P16R (60°C, 30 PCRcycles). In wild type, a 220 bp band is obtained while a 180 bp band is obtained for the mutant allele.

For *hei10-2*, the left border of the T-DNA insertion was amplified by PCR with primers T3F20-P5 and LbSALK2 while the wild-type allele was amplified with primers T3F20-P5 and T3F20-P6. The annealing step was performed at 60°C, the expected size for the mutant allele was 970 bp; for the wild-type allele is 1070 bp.

For *hei10-4*, the wild-type allele was amplified with primers At1g53470-P5 and T3F20-P7 (58°C, 30 PCRcycles, 847 bp). The mutant allele was amplified with primers At1g53470-P5 and At1g53510-P4. (58°C, 30 PCRcycles, 434 bp).

For the *msh4^Col^* mutant, the wild-type allele was amplified using primers 636296U and 636296L while the mutant allele was amplified using primers 636296L and LbSalk2.

*msh4^W^* ^s^ was genotyped using primers msh4#1 and msh4#2 at 57°C for 30 cycles (wt allele: 805 bp; mutant allele 749 bp).

*mer3^W^* ^s^ was genotyped using primers mer3#36 and mer3#37 at 57°C for 30 cycles (wt allele: 176 bp; mutant allele 156 bp).

*mlh1^Col^* was genotyped using primers sk25975u and sk25975l for the wild-type allele (1000 bp at 57°C) and sk25975l and PSK tail 1 for the mutant allele (700 bp).

*rad51^Col^* was genotyped using primers RAD51-fw and RAD51-Rev for the wild-type allele (994 bp at 65°C) and RAD51-fw and LbGABI1 for the mutant allele (700 bp).

### Genetic analyses

#### Allelelism tests

We crossed semi-sterile *hei10-1*, *hei10-3*, *hei10-4* and *hei10-5* mutants (two plants) with a fertile plant heterozygous for the *hei10-2* mutation. The resulting F1 progeny displayed the low-fertility phenotype in a 50∶50 ratio, demonstrating that they were allelic. We also crossed heterozygotes for the mutations *hei10-5* and *hei10-4*, as well as heterozygotes for *hei10-1* and *hei10-4* and heterozygotes for mutations *hei10-5* and *hei10-1* and checked that one quarter of the progeny displayed a low fertility phenotype.

#### Interference measurements

We used the three FTL lines 1273, 1659 and 993 corresponding respectively to a DsRED marker in position 18,164,269 bp (allele R), a eYFP marker in position 23,080567 bp (allele Y) and a eCFP in position 25,731,311 bp (allele C) on chromosome 5. These three markers define the I5a and I5b intervals described in Francis 2007 and Berchowitz 2008. We produced plants *qrt^−/−^ hei10-2^−/+^ and qrt^−/−^ hei10-2^−/+^RYC/RYC*. We crossed these two plants and in the progeny analysed tetrad fluorescence of semi-sterile plants *qrt^−/−^ hei10-2^−/−^RYC/+++* or fertile plants either *qrt^−/−^ hei10-2^−/+^*RYC/+++ or *qrt^−/−^ hei10-2^+/+^*RYC/+++. Plants were grown in growth chambers. Tetrad analyses were carried out as described in [42], on the major inflorescence.

#### Double mutant generation

All double mutants were obtained by crossing plants which were heterozygous for each mutation. The resulting hybrids were self-pollinated. PCR screening was then used to identify plants in the F2 progeny that were homozygous for both mutations.

### cDNA studies

The full length cDNA clone corresponding to At1g53490 (accession number BX814259) was obtained from INRA-CNRGV (http://cnrgv.toulouse.inra.fr). For RT-PCR experiments, cDNA were obtained from Col-0 flower buds, roots or leaf tissues as described in [83], then normalised according to the expression of the phosphoribosyltransferase-encoding gene (APT, [84]), after 30 amplification cycles at 60°C. *HEI10* amplification was obtained after two rounds of nested PCR, either first with primers P16 and P5R (25 cycles at 65°C), then with primers P18 and P10 (25 cycles at 65°C) or first with primers P16 and P17 (25 cycles at 65°C), and second with primers P18 and P19 (25 cycles at 65°C).

### Antibodies

The anti-ASY1 polyclonal antibody was described by [45]. It was used at a dilution of 1∶500. The anti-ZYP1 polyclonal antibody was described by [10]. It was used at a dilution of 1∶500.

The anti-DMC1 antibody was described in [32] and the MLH1 antibody in [47]. They were used at a dilution of 1∶20 and 1∶200 respectively.

The anti-HEI10 antibody was generated as follows: the full length cDNA fragment was amplified with 5′*Bam*HI primer (TAGAggatccCatgAGATGCA) and 3′*Xho*I primer (TAAGctcgagCGtGAACaGCT). The fragment was inserted into pTOPO2-1 (Invitrogen) and sequenced. A *Bam*HI-*Xho*I fragment containing the full-length cDNA sequence was then subcloned in-frame into pET29b (Novagen). The resulting construct was transferred to *E. coli* Rosetta 2 cells (Novagen) and over-expressed upon induction for 6 days at 4°C. the recombinant protein fusion was purified as described in [47] and sent to Eurogentec to produce polyclonal rabbit antibodies. Anti-HEI10 antibodies were affinity-purified as described in [47] and used at a dilution of 1∶200.

### Microscopy

Comparison of the early stages of microsporogenesis and the development of PMCs was carried out as described in [69]. Preparation of prophase stage spreads for immunocytology was performed according to [45] with the modifications described in [47], [85].

Chiasma number was estimated on metaphase I spread PMC chromosomes counterstained with DAPI based on bivalent configuration as described in [41]: a rod bivalent was considered to contain a single chiasma, while a ring bivalent was recorded as two (one on each arm).

Observations were made using a Leica (http://www.leica.com) DM RXA2 microscope or a Zeiss (http://www.zeiss.fr) Axio Imager 2 microscope; photographs were taken using a CoolSNAP HQ (Roper, http://www.roperscientific.com) camera driven by OpenLAB 4.0.4 software or a Zeiss camera AxioCam MR driven by Axiovision 4.7. All images were further processed with OpenLAB 4.0.4, Axiovision 4.7, or AdobePhotoshop 7.0 (http://www.adobe.com).

### Bioinformatics studies

A BLAST similarity network was constructed. In a first step all candidate Zip3-like and HEI10 sequences for a predetermined set of species were obtained (nodes in Figure 2B). The set was obtained using NCBI-BLAST with the HEI10 orthologues and PTHR22663 family members as the query on NCBI-nr limited to the predefined set of organisms as the subject database (CAST-masking was applied prior to all BLAST searches, significance threshold 0.01). In a second step the BLAST similarities within the sequence set were determined (edges in Figure 2B). The preselected set of sequences was reapplied to the above NCBI-BLAST procedure. The BLAST similarity network was visualized in Cytoscape [86].

### Statistical analyses

Mean chiasma frequencies in various genotypes were compared using one-way analysis of variance (ANOVA) followed by a Student-Newman-Keuls (SNK) test (proc GLM; SAS Institute Inc., 1999).

### Primer sequences

P6: CTTATGATCCTTGTAGGTAGT; P10: TGCTTGCATACCCTCACAT; P16: CTCAGTAATGATGGGGCATGTC; P17:TACGTGAACAGCTGAGGGCG; P18: TGGGGCATGTCCCATTTGTGA; P19: TGAGGGCGGGAACGAGAGAG; P21: GCGTAGCAGCTTGAGATACT; P5R: TGCTGTATGGACCTGCTCC; P19R: CTCTCTCGTTCCCGCCCTCA; T3F20-P6: CTTATGATCCTTGTAGGTAGT; T3F20-P5: GGAGCAGGTCCATACAGCA; At1g53470-P2: CACAAGCCCTAGCCATAGA; At1g53470-P1: CCTCTGTCGTCAACGGCAGT; T3F20-P9: CATCCAACGTAGACTTGCAT; T3F20-P8: TGGTAGTGGTGGCTCAGTGT; At1g53510-P1: TGGAGTTGTGTGTGCAGCTA; At1g53510-P2: GGGAGAGTTCAAGTCATCCCT; T3F20-P5: GGAGCAGGTCCATACAGCA; T3F20-P17: TACGTGAACAGCTGAGGGCG; 636296U: CTTCTTGCAGGTTGTGTTTG; 636296L: GCCAGCTGTTTTTGTTGTC; msh4#1 : TTATTTGTACTGCTTGGCAA; msh4#2 : GGATCATAGAAACGCAACA; mer3#36 : ATTCATGACTCCTATCACTTG; mer3#37 : AGAAGAGTTGAGCTAAGAGTTC; sk25975u : AAGTTTGTGAGGCATCATCATG; sk25975l : TTGCAGATTCAATACAACAACAC; RAD51-fw : ATGCCAAGGTTGACAAGATTG; RAD51-Rev : CTCCCCTTCCAGAGAAATCTG; APT-RT1: TCCCAGAATCGCTAAGATTGCC; APT-RT2-1: CTCAATTACGCAAGCAC; LbSALK2: GCTTTCTTCCCTTCCTTTCTC; PSK tail 1: TTCTCATCTAAGCCCCCATTTGG; LbGABI1: CCCATTTGGACGTGAATGTAGACAC.

## Supporting Information

Figure S1 *HEI10* expression in *hei10* mutants. RT-PCR on cDNA isolated from flower buds from the five mutant lines and wild-type plants (Ws and Col-0 accessions). *HEI10* expression was followed after two rounds of PCR primers P16 and P5R followed by primers P18 and P10 or P16 and P17 followed by P18 and P19. *APT* expression [84] was used to normalise the various cDNA samples. L: Fermentas 1 Kb DNA ladder; *Δ*1: water control for the first round of PCR, *Δ*2: water control for nested PCR.
(DOCX)

Figure S2 Molecular characterisation of the *hei10-1* allele. A: Aberrant transcripts are produced in *hei10-1*. RT-PCR on flower buds from the *hei10-1* mutant line or wild-type plants (Ws) are shown as well as the analyses of the *hei10-1* RT-PCR products cloned and sequenced. B: Predicted HEI10 protein in wild type and *hei10-1*. Wild-type HEI10 protein sequence (in black) is compared to the predicted translational products issued of the cDNA variants described in A.
(DOCX)

Figure S3 *hei10-4* genomic region. Schematic representation of At1g53490 genomic region as predicted in TAIR10 (http://www.arabidopsis.org/). Amplification between primers 470P3 and 500-P1R generated a band in the *hei10-4* mutant only. Sequencing of this amplification product revealed that nucleotides 19,962,396 to19,971,065 are absent in *hei10-4*.
(DOCX)

Figure S4 *hei10-5* genomic region. Schematic representation of At1g53490 genomic region as predicted in TAIR10 (http://www.arabidopsis.org/). Predicted ORF as well as primers used for *hei10-5* deletion characterisation are shown.
(DOCX)

Figure S5 *HEI10* expression in different tissues. RT-PCR on cDNA isolated from leaves (Le), roots (R) and flower buds (B) of WS-4 wild-type plants were calibrated according to the expression of the phosphoribosyltransferase-encoding gene (APT, [84]) in A. They were then used to detect *HEI10* expression after two rounds of nested PCR, first with primers P16 and P17, and second with primers P18 and P19. L: Fermentas 1 Kb DNA ladder.
(DOCX)

Figure S6 *hei10* mutants show low fertility and defects in male sporogenesis. A: Comparison of wild-type (Wt) and homozygous *hei10-1* (*hei10*) mutant plants after a month in the greenhouse. Arrows show siliques that elongate in wild type but not in mutant. B: Meiotic products after anther clearing of wild type (wt) and *hei10-1* mutant show abnormal male sporogenesis in *hei10*.
(DOCX)

Figure S7 HEI10 can be detected on chromosomes throughout the entire meiotic prophase. Co-immunolocalisation of ASY1 and HEI10 on wild-type Col-0 plants after lipsol spreading of PMC chromosomes. A: Leptotene, B: Early Zygotene, C–E: Pachytene, F: Diakinisis. Arrows in B indicate a region that is likely synapsed as suggested by the faint ASY1 signal. Bar: 10 µm.
(TIF)

Figure S8 HEI10 co-localisation with SC components. A–D: Co-immunolocalisation of HEI10 (red) and the central element of the SC (ZYP1, green). E–H: Co-immunolocalisation of HEI10 (green) with ASY1 (component of the meiotic chromosome axis).
(TIF)

Figure S9 Co-immunolocalisation of ASY1 and HEI10 in *hei10* mutants (Acetic Acid Spreads). Co-immunolocalisation of ASY1 and HEI10 on PMC chromosomes after acetic acid spreading. A–E: *hei10-1* mutant (Ws-4 background). F–J: *hei10-2* mutant (Col-0 background). For each cell the three merged signals are shown (DAPI in white, ASY1 in red, and HEI10 in green). A, F: Early Zygotene, B, G: Late Zygotene, C, H, I: Pachytene, D: Diplotene, E, J: Diakinesis. Arrows on A and B indicate synapsed regions, while arrows on E indicate chiasmata HEI10 labelled. Bar: 10 µm.
(TIF)

Figure S10 Co-immunolocalisation of ASY1 and HEI10 in *hei10* mutants (Lipsol Spreads). PMC were lipsol-spread, then immunolabelled with anti-ASY1 (red) and anti-HEI10 (green) antibodies. A: Leptotene, B: Zygotene, C–F: Pachytene.
(TIF)

## References

[1] Keeney S, Giroux CN, Kleckner N (1997). Meiosis-specific DNA double-strand breaks are catalyzed by Spo11, a member of a widely conserved protein family.. Cell.

[2] Bishop DK, Zickler D (2004). Early decision; meiotic crossover interference prior to stable strand exchange and synapsis.. Cell.

[3] Muller HJ (1916). The mechanisms of crossing-over.. American Naturalist.

[4] Whitby MC (2005). Making crossovers during meiosis.. Biochem Soc Trans.

[5] Osman K, Higgins JD, Sanchez-Moran E, Armstrong SJ, Franklin FC (2011). Pathways to meiotic recombination in Arabidopsis thaliana.. New Phytol.

[6] Berchowitz LE, Francis KE, Bey AL, Copenhaver GP (2007). The Role of AtMUS81 in Interference-Insensitive Crossovers in A. thaliana.. PLoS Genet.

[7] De Los Santos T, Hunter N, Lee C, Larkin B, Loidl J (2003). The mus81/mms4 endonuclease acts independently of double-holliday junction resolution to promote a distinct subset of crossovers during meiosis in budding yeast.. Genetics.

[8] Higgins JD, Buckling EF, Franklin FC, Jones GH (2008). Expression and functional analysis of AtMUS81 in Arabidopsis meiosis reveals a role in the second pathway of crossing-over.. Plant J.

[9] Lynn A, Soucek R, Borner GV (2007). ZMM proteins during meiosis: Crossover artists at work.. Chromosome Res.

[10] Higgins JD, Armstrong SJ, Franklin FC, Jones GH (2004). The Arabidopsis MutS homolog AtMSH4 functions at an early step in recombination: evidence for two classes of recombination in Arabidopsis.. Genes Dev.

[11] Higgins JD, Vignard J, Mercier R, Pugh AG, Franklin FC (2008). AtMSH5 partners AtMSH4 in the class I meiotic crossover pathway in Arabidopsis thaliana, but is not required for synapsis.. Plant J.

[12] Kneitz B, Cohen PE, Avdievich E, Zhu L, Kane MF (2000). MutS homolog 4 localization to meiotic chromosomes is required for chromosome pairing during meiosis in male and female mice.. Genes Dev.

[13] Santucci-Darmanin S, Walpita D, Lespinasse F, Desnuelle C, Ashley T (2000). MSH4 acts in conjunction with MLH1 during mammalian meiosis.. Faseb J.

[14] de Boer E, Stam P, Dietrich AJ, Pastink A, Heyting C (2006). Two levels of interference in mouse meiotic recombination.. Proc Natl Acad Sci U S A.

[15] Moens PB, Kolas NK, Tarsounas M, Marcon E, Cohen PE (2002). The time course and chromosomal localization of recombination-related proteins at meiosis in the mouse are compatible with models that can resolve the early DNA-DNA interactions without reciprocal recombination.. J Cell Sci.

[16] Neyton S, Lespinasse F, Moens PB, Paul R, Gaudray P (2004). Association between MSH4 (MutS homologue 4) and the DNA strand-exchange RAD51 and DMC1 proteins during mammalian meiosis.. Mol Hum Reprod.

[17] Baker SM, Plug AW, Prolla TA, Bronner CE, Harris AC (1996). Involvement of mouse Mlh1 in DNA mismatch repair and meiotic crossing over.. Nat Genet.

[18] Hunter N, Borts RH (1997). Mlh1 is unique among mismatch repair proteins in its ability to promote crossing-over during meiosis.. Genes Dev.

[19] Jackson N, Sanchez-Moran E, Buckling E, Armstrong SJ, Jones GH (2006). Reduced meiotic crossovers and delayed prophase I progression in AtMLH3-deficient Arabidopsis.. Embo J.

[20] Kolas NK, Svetlanov A, Lenzi ML, Macaluso FP, Lipkin SM (2005). Localization of MMR proteins on meiotic chromosomes in mice indicates distinct functions during prophase I.. J Cell Biol.

[21] Lipkin SM, Moens PB, Wang V, Lenzi M, Shanmugarajah D (2002). Meiotic arrest and aneuploidy in MLH3-deficient mice.. Nat Genet.

[22] Page SL, Hawley RS (2004). The genetics and molecular biology of the synaptonemal complex.. Annu Rev Cell Dev Biol.

[23] de Boer E, Heyting C (2006). The diverse roles of transverse filaments of synaptonemal complexes in meiosis.. Chromosoma.

[24] Agarwal S, Roeder GS (2000). Zip3 provides a link between recombination enzymes and synaptonemal complex proteins.. Cell.

[25] Chua PR, Roeder GS (1998). Zip2, a meiosis-specific protein required for the initiation of chromosome synapsis.. Cell.

[26] Tsubouchi T, Zhao H, Roeder GS (2006). The meiosis-specific zip4 protein regulates crossover distribution by promoting synaptonemal complex formation together with zip2.. Dev Cell.

[27] Shinohara M, Oh SD, Hunter N, Shinohara A (2008). Crossover assurance and crossover interference are distinctly regulated by the ZMM proteins during yeast meiosis.. Nat Genet.

[28] de Vries SS, Baart EB, Dekker M, Siezen A, de Rooij DG (1999). Mouse MutS-like protein Msh5 is required for proper chromosome synapsis in male and female meiosis.. Genes Dev.

[29] Chen C, Zhang W, Timofejeva L, Gerardin Y, Ma H (2005). The Arabidopsis ROCK-N-ROLLERS gene encodes a homolog of the yeast ATP-dependent DNA helicase MER3 and is required for normal meiotic crossover formation.. Plant J.

[30] Mercier R, Jolivet S, Vezon D, Huppe E, Chelysheva L (2005). Two meiotic crossover classes cohabit in Arabidopsis: one is dependent on MER3,whereas the other one is not.. Curr Biol.

[31] Wang K, Tang D, Wang M, Lu J, Yu H (2009). MER3 is required for normal meiotic crossover formation, but not for presynaptic alignment in rice.. J Cell Sci.

[32] Chelysheva L, Gendrot G, Vezon D, Doutriaux MP, Mercier R (2007). Zip4/Spo22 is required for class I CO formation but not for synapsis completion in Arabidopsis thaliana.. PLoS Genet.

[33] Mazina OM, Mazin AV, Nakagawa T, Kolodner RD, Kowalczykowski SC (2004). Saccharomyces cerevisiae Mer3 helicase stimulates 3′-5′ heteroduplex extension by Rad51; implications for crossover control in meiotic recombination.. Cell.

[34] Snowden T, Acharya S, Butz C, Berardini M, Fishel R (2004). hMSH4-hMSH5 recognizes Holliday Junctions and forms a meiosis-specific sliding clamp that embraces homologous chromosomes.. Mol Cell.

[35] Macaisne N, Vignard J, Mercier R (2011). SHOC1 and PTD form an XPF-ERCC1-like complex that is required for formation of class I crossovers.. J Cell Sci.

[36] Macaisne N, Novatchkova M, Peirera L, Vezon D, Jolivet S (2008). SHOC1, an XPF endonuclease-related protein, is essential for the formation of class I meiotic crossovers.. Curr Biol.

[37] Perry J, Kleckner N, Borner GV (2005). Bioinformatic analyses implicate the collaborating meiotic crossover/chiasma proteins Zip2, Zip3, and Spo22/Zip4 in ubiquitin labeling.. Proc Natl Acad Sci U S A.

[38] Cheng CH, Lo YH, Liang SS, Ti SC, Lin FM (2006). SUMO modifications control assembly of synaptonemal complex and polycomplex in meiosis of Saccharomyces cerevisiae.. Genes Dev.

[39] Ward JO, Reinholdt LG, Motley WW, Niswander LM, Deacon DC (2007). Mutation in mouse hei10, an e3 ubiquitin ligase, disrupts meiotic crossing over.. PLoS Genet.

[40] Ross KJ, Fransz P, Jones GH (1996). A light microscopic atlas of meiosis in Arabidopsis thaliana.. Chromosome Res.

[41] Sanchez Moran E, Armstrong SJ, Santos JL, Franklin FC, Jones GH (2001). Chiasma formation in Arabidopsis thaliana accession Wassileskija and in two meiotic mutants.. Chromosome Res.

[42] Berchowitz LE, Copenhaver GP (2008). Fluorescent Arabidopsis tetrads: a visual assay for quickly developing large crossover and crossover interference data sets.. Nat Protoc.

[43] Perkins DD (1949). Biochemical mutants in the smut fungus Ustilago maydis.. Genetics.

[44] Li W, Chen C, Markmann-Mulisch U, Timofejeva L, Schmelzer E (2004). The Arabidopsis AtRAD51 gene is dispensable for vegetative development but required for meiosis.. Proc Natl Acad Sci U S A.

[45] Armstrong SJ, Caryl AP, Jones GH, Franklin FC (2002). Asy1, a protein required for meiotic chromosome synapsis, localizes to axis-associated chromatin in Arabidopsis and Brassica.. J Cell Sci.

[46] Lhuissier FG, Offenberg HH, Wittich PE, Vischer NO, Heyting C (2007). The mismatch repair protein MLH1 marks a subset of strongly interfering crossovers in tomato.. Plant Cell.

[47] Chelysheva L, Grandont L, Vrielynck N, le Guin S, Mercier R (2010). An easy protocol for studying chromatin and recombination protein dynamics during Arabidopsis thaliana meiosis: immunodetection of cohesins, histones and MLH1.. Cytogenet Genome Res.

[48] Higgins JD, Sanchez-Moran E, Armstrong SJ, Jones GH, Franklin FC (2005). The Arabidopsis synaptonemal complex protein ZYP1 is required for chromosome synapsis and normal fidelity of crossing over.. Genes Dev.

[49] Punta M, Coggill PC, Eberhardt RY, Mistry J, Tate J (2012). The Pfam protein families database.. Nucleic acids research.

[50] Delorenzi M, Speed T (2002). An HMM model for coiled-coil domains and a comparison with PSSM-based predictions.. Bioinformatics.

[51] Altenhoff AM, Dessimoz C (2009). Phylogenetic and functional assessment of orthologs inference projects and methods.. PLoS Comput Biol.

[52] Sayers EW, Barrett T, Benson DA, Bolton E, Bryant SH (2012). Database resources of the National Center for Biotechnology Information.. Nucleic acids research.

[53] Mi H, Dong Q, Muruganujan A, Gaudet P, Lewis S (2010). PANTHER version 7: improved phylogenetic trees, orthologs and collaboration with the Gene Ontology Consortium.. Nucleic acids research.

[54] Wijeratne AJ, Chen C, Zhang W, Timofejeva L, Ma H (2006). The Arabidopsis thaliana PARTING DANCERS gene encoding a novel protein is required for normal meiotic homologous recombination.. Mol Biol Cell.

[55] Singh MK, Nicolas E, Gherraby W, Dadke D, Lessin S (2007). HEI10 negatively regulates cell invasion by inhibiting cyclin B/Cdk1 and other promotility proteins.. Oncogene.

[56] Toby GG, Gherraby W, Coleman TR, Golemis EA (2003). A novel RING finger protein, human enhancer of invasion 10, alters mitotic progression through regulation of cyclin B levels.. Mol Cell Biol.

[57] Smith AP, Weeraratna AT, Spears JR, Meltzer PS, Becker D (2004). SAGE identification and fluorescence imaging analysis of genes and transcripts in melanomas and precursor lesions.. Cancer Biol Ther.

[58] Edelmann W, Cohen PE, Kane M, Lau K, Morrow B (1996). Meiotic pachytene arrest in MLH1-deficient mice.. Cell.

[59] Woods LM, Hodges CA, Baart E, Baker SM, Liskay M (1999). Chromosomal influence on meiotic spindle assembly: abnormal meiosis I in female Mlh1 mutant mice.. J Cell Biol.

[60] Edelmann W, Cohen PE, Kneitz B, Winand N, Lia M (1999). Mammalian MutS homologue 5 is required for chromosome pairing in meiosis.. Nat Genet.

[61] Strong ER, Schimenti JC (2010). Evidence Implicating CCNB1IP1, a RING Domain-Containing Protein Required for Meiotic Crossing Over in Mice, as an E3 SUMO Ligase.. Genes (Basel).

[62] Baudat F, de Massy B (2007). Regulating double-stranded DNA break repair towards crossover or non-crossover during mammalian meiosis.. Chromosome Res.

[63] De Muyt A, Mercier R, Mezard C, Grelon M (2009). Meiotic recombination and crossovers in plants.. Genome Dyn.

[64] Moens PB, Chen DJ, Shen Z, Kolas N, Tarsounas M (1997). Rad51 immunocytology in rat and mouse spermatocytes and oocytes.. Chromosoma.

[65] Osman K, Sanchez-Moran E, Mann SC, Jones GH, Franklin FC (2009). Replication protein A (AtRPA1a) is required for class I crossover formation but is dispensable for meiotic DNA break repair.. Embo J.

[66] Storlazzi A, Gargano S, Ruprich-Robert G, Falque M, David M (2010). Recombination proteins mediate meiotic spatial chromosome organization and pairing.. Cell.

[67] Bhalla N, Wynne DJ, Jantsch V, Dernburg AF (2008). ZHP-3 acts at crossovers to couple meiotic recombination with synaptonemal complex disassembly and bivalent formation in C. elegans.. PLoS Genet.

[68] Tsubouchi T, Macqueen AJ, Roeder GS (2008). Initiation of meiotic chromosome synapsis at centromeres in budding yeast.. Genes Dev.

[69] Grelon M, Vezon D, Gendrot G, Pelletier G (2001). AtSPO11-1 is necessary for efficient meiotic recombination in plants.. Embo J.

[70] Dernburg AF, McDonald K, Moulder G, Barstead R, Dresser M (1998). Meiotic recombination in C. elegans initiates by a conserved mechanism and is dispensable for homologous chromosome synapsis.. Cell.

[71] Kong A, Thorleifsson G, Stefansson H, Masson G, Helgason A (2008). Sequence variants in the RNF212 gene associate with genome-wide recombination rate.. Science.

[72] Carpenter AT (1975). Electron microscopy of meiosis in Drosophila melanogaster females: II. The recombination nodule–a recombination-associated structure at pachytene?. Proc Natl Acad Sci U S A.

[73] Zickler D, Kleckner N (1999). Meiotic chromosomes: integrating structure and function.. Annu Rev Genet.

[74] Anderson LK, Stack SM (2005). Recombination nodules in plants.. Cytogenet Genome Res.

[75] Guillon H, de Massy B (2002). An initiation site for meiotic crossing-over and gene conversion in the mouse.. Nat Genet.

[76] Padmore R, Cao L, Kleckner N (1991). Temporal comparison of recombination and synaptonemal complex formation during meiosis in S. cerevisiae.. Cell.

[77] Zickler D (1977). Development of the synaptonemal complex and the “recombination nodules” during meiotic prophase in the seven bivalents of the fungus Sordaria macrospora Auersw.. Chromosoma.

[78] Gillies CB (1979). The Relationship between Synaptinemal Complexes, Recombination Nodules and Crossing over in NEUROSPORA CRASSA Bivalents and Translocation Quadrivalents.. Genetics.

[79] Leflon M, Grandont L, Eber F, Huteau V, Coriton O (2010). Crossovers get a boost in Brassica allotriploid and allotetraploid hybrids.. Plant Cell.

[80] Bechtold N, Ellis J, Pelletier G (1993). In Planta, Agrobacterium mediated gene transfer by integration of adult Arabidopsis thaliana plants.. C R Acad Sci Paris.

[81] Alonso JM, Stepanova AN, Leisse TJ, Kim CJ, Chen H (2003). Genome-wide insertional mutagenesis of Arabidopsis thaliana.. Science.

[82] De Muyt A, Pereira L, Vezon D, Chelysheva L, Gendrot G (2009). A high throughput genetic screen identifies new early meiotic recombination functions in Arabidopsis thaliana.. PLoS Genet.

[83] De Muyt A, Vezon D, Gendrot G, Gallois JL, Stevens R (2007). AtPRD1 is required for meiotic double strand break formation in Arabidopsis thaliana.. Embo J.

[84] Moffatt BA, McWhinnie EA, Agarwal SK, Schaff DA (1994). The adenine phosphoribosyltransferase-encoding gene of Arabidopsis thaliana.. Gene.

[85] Chelysheva L, Diallo S, Vezon D, Gendrot G, Vrielynck N (2005). AtREC8 and AtSCC3 are essential to the monopolar orientation of the kinetochores during meiosis.. J Cell Sci.

[86] Smoot ME, Ono K, Ruscheinski J, Wang PL, Ideker T (2011). Cytoscape 2.8: new features for data integration and network visualization.. Bioinformatics.

[87] Edgar RC (2004). MUSCLE: a multiple sequence alignment method with reduced time and space complexity.. BMC Bioinformatics.

[88] Jantsch V, Pasierbek P, Mueller MM, Schweizer D, Jantsch M (2004). Targeted gene knockout reveals a role in meiotic recombination for ZHP-3, a Zip3-related protein in Caenorhabditis elegans.. Mol Cell Biol.

