# Supplementary figures and images for: The Arabidopsis HEI10 Is a New ZMM Protein Related to Zip3
Source: PLoS Genet. 2012 Jul 26;8(7):e1002799. doi: 10.1371/journal.pgen.1002799 (PMC3405992; doi:10.1371/journal.pgen.1002799)

**Figure S1: *HEI10* expression in *hei10* mutants**


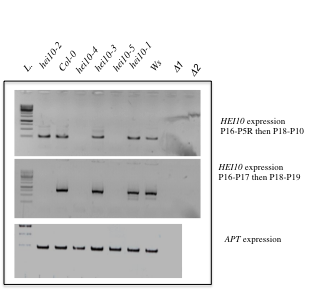

Supplement: Figure S1 — HEI10 expression in hei10 mutants. RT-PCR on cDNA isolated from flower buds from the five mutant lines and wild-type plants (Ws and Col-0 accessions). HEI10 expression was followed after two rounds of PCR primers P16 and P5R followed by primers P18 and P10 or P16 and P17 followed by P18 and P19. APT expression [84] was used to normalise the various cDNA samples. L: Fermentas 1 Kb DNA ladder; Δ1: water control for the first round of PCR, Δ2: water control for nested PCR. (DOCX) [file pgen.1002799.s001.docx]

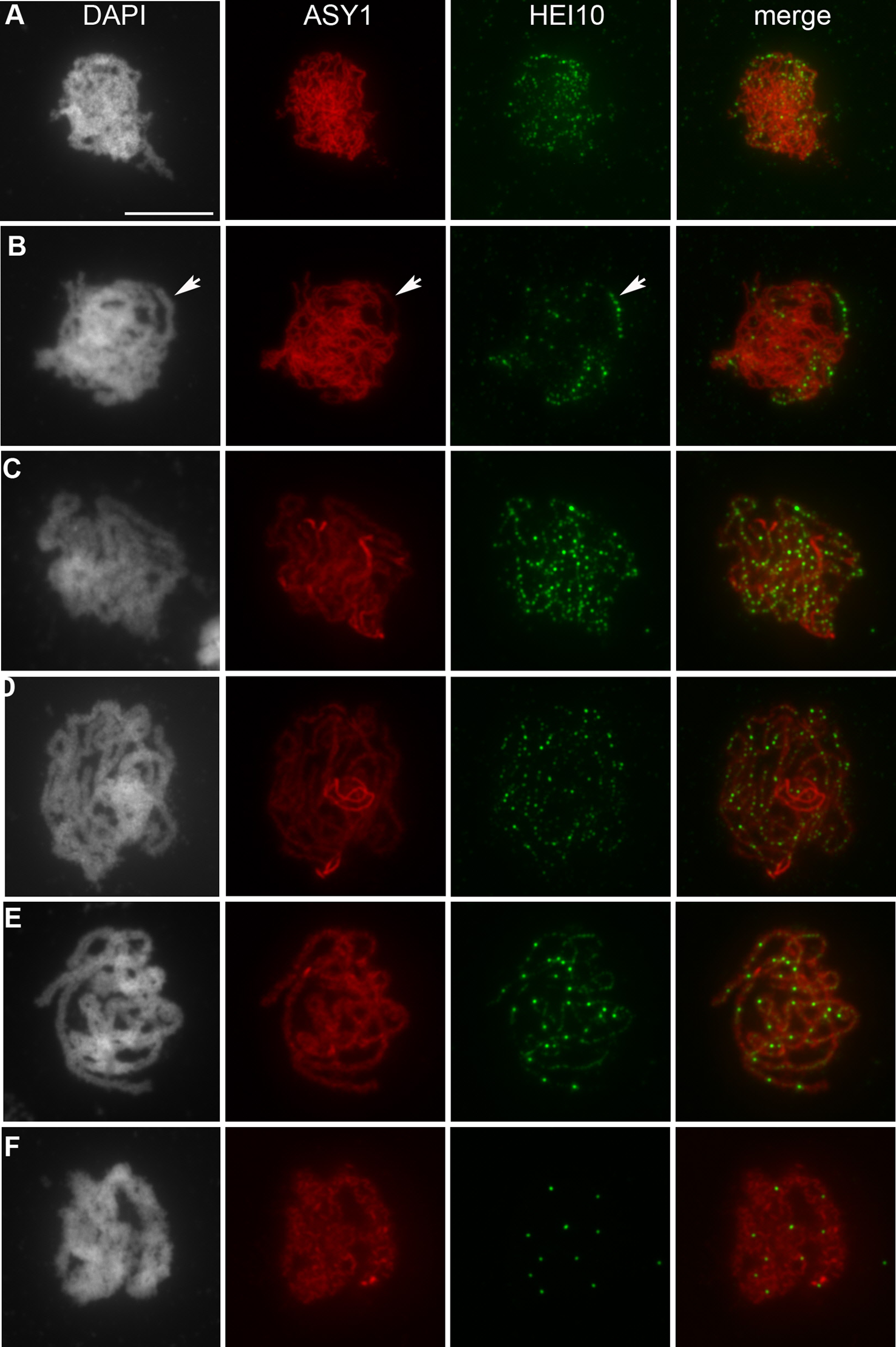

Supplement: Figure S7 — HEI10 can be detected on chromosomes throughout the entire meiotic prophase. Co-immunolocalisation of ASY1 and HEI10 on wild-type Col-0 plants after lipsol spreading of PMC chromosomes. A: Leptotene, B: Early Zygotene, C–E: Pachytene, F: Diakinisis. Arrows in B indicate a region that is likely synapsed as suggested by the faint ASY1 signal. Bar: 10 µm. (TIF) [file pgen.1002799.s007.tif]

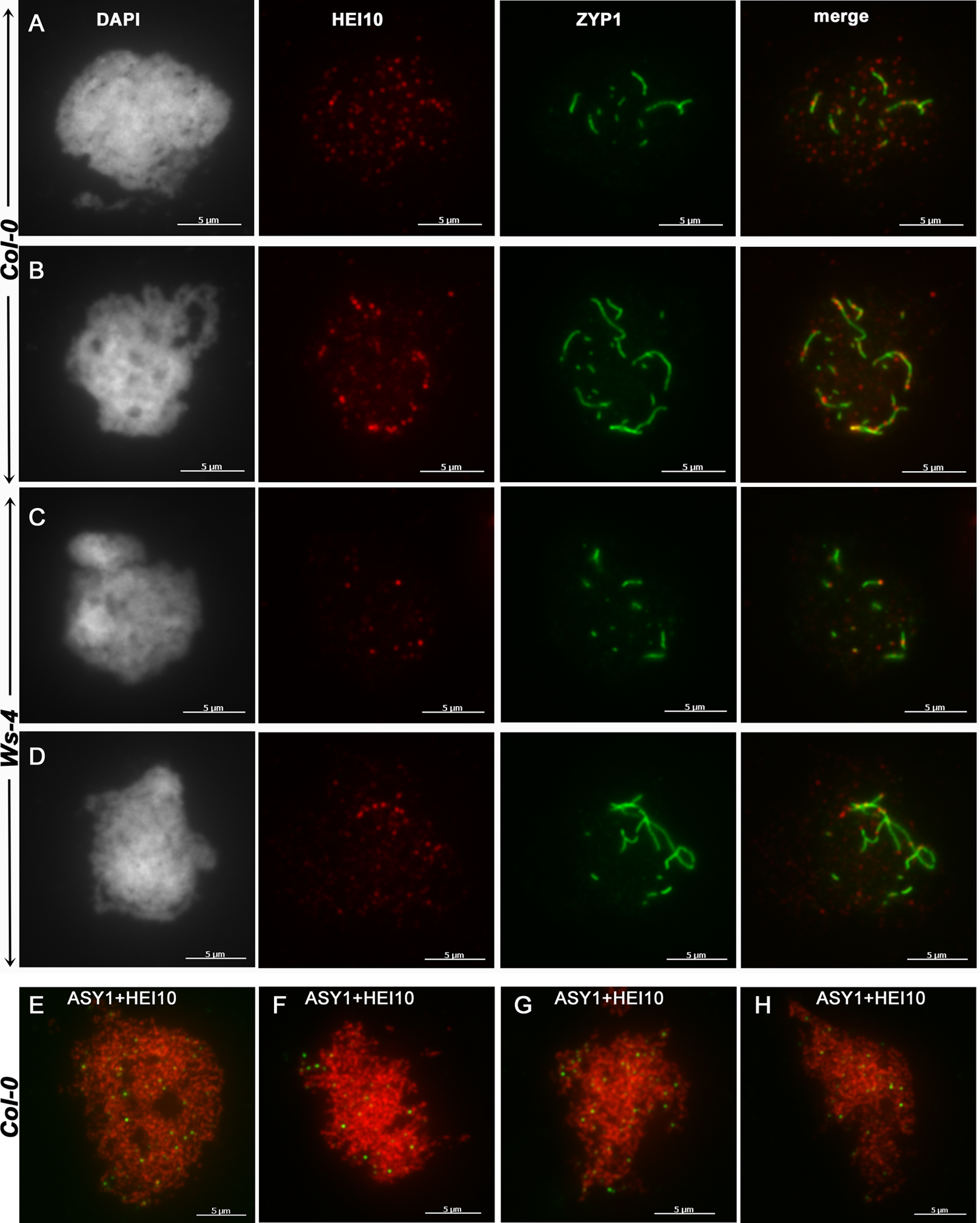

Supplement: Figure S8 — HEI10 co-localisation with SC components. A–D: Co-immunolocalisation of HEI10 (red) and the central element of the SC (ZYP1, green). E–H: Co-immunolocalisation of HEI10 (green) with ASY1 (component of the meiotic chromosome axis). (TIF) [file pgen.1002799.s008.tif]

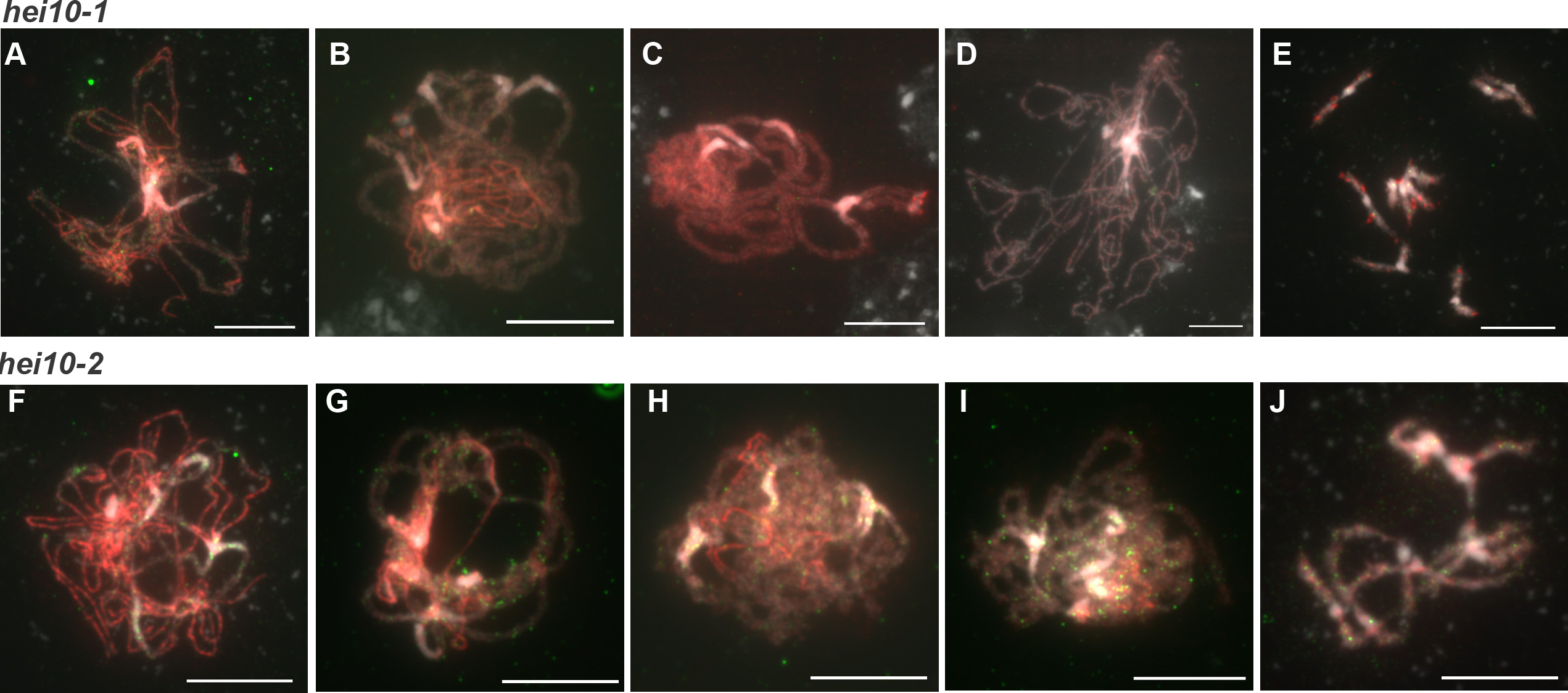

Supplement: Figure S9 — Co-immunolocalisation of ASY1 and HEI10 in hei10 mutants (Acetic Acid Spreads). Co-immunolocalisation of ASY1 and HEI10 on PMC chromosomes after acetic acid spreading. A–E: hei10-1 mutant (Ws-4 background). F–J: hei10-2 mutant (Col-0 background). For each cell the three merged signals are shown (DAPI in white, ASY1 in red, and HEI10 in green). A, F: Early Zygotene, B, G: Late Zygotene, C, H, I: Pachytene, D: Diplotene, E, J: Diakinesis. Arrows on A and B indicate synapsed regions, while arrows on E indicate chiasmata HEI10 labelled. Bar: 10 µm. (TIF) [file pgen.1002799.s009.tif]

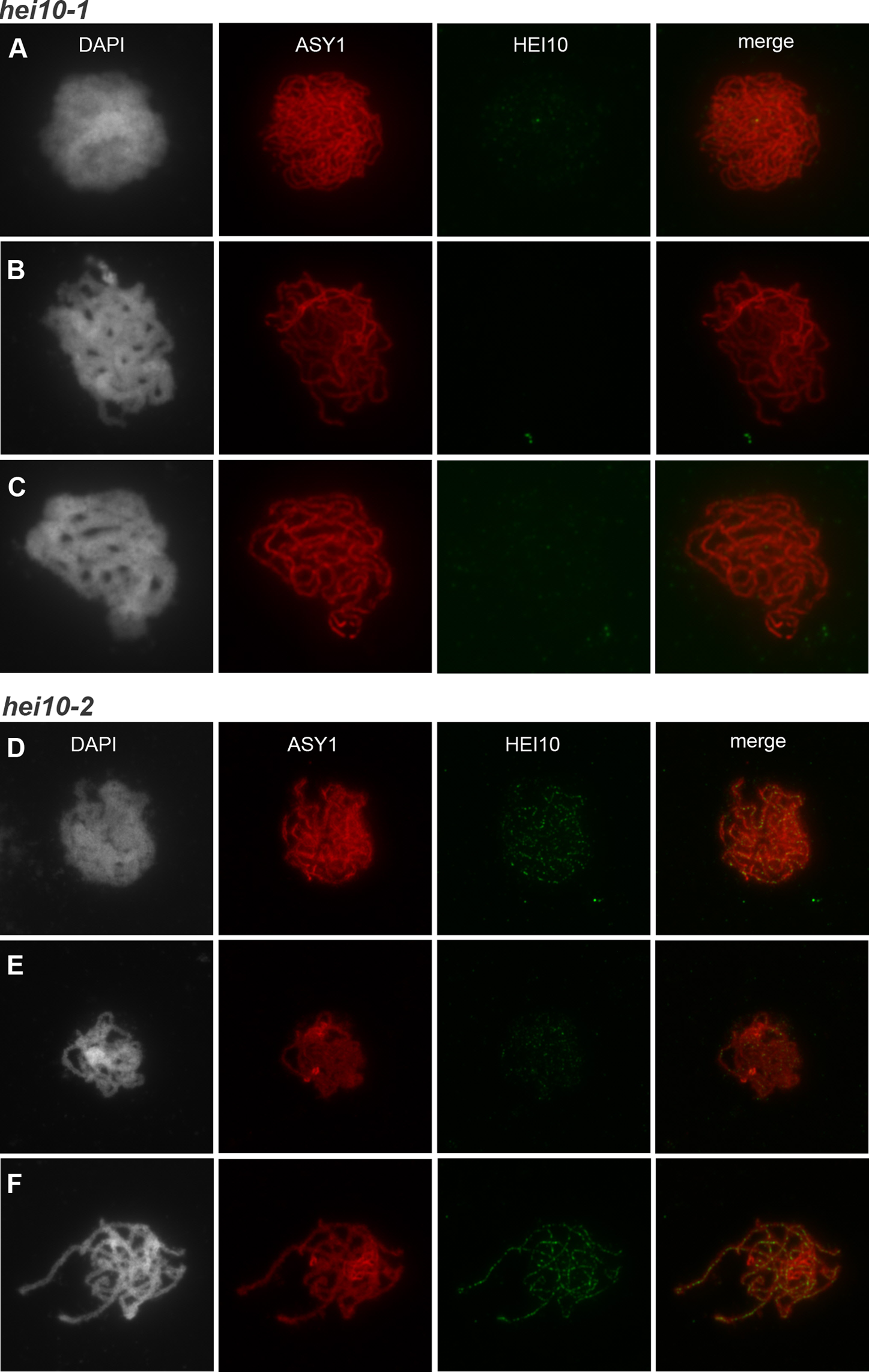

Supplement: Figure S10 — Co-immunolocalisation of ASY1 and HEI10 in hei10 mutants (Lipsol Spreads). PMC were lipsol-spread, then immunolabelled with anti-ASY1 (red) and anti-HEI10 (green) antibodies. A: Leptotene, B: Zygotene, C–F: Pachytene. (TIF) [file pgen.1002799.s010.tif]
