# Supplementary material for: The Arabidopsis HEI10 Is a New ZMM Protein Related to Zip3
Source: PLoS Genet. 2012 Jul 26;8(7):e1002799. doi: 10.1371/journal.pgen.1002799 (PMC3405992; doi:10.1371/journal.pgen.1002799)
Supplement: Figure S2 — Molecular characterisation of the hei10-1 allele. A: Aberrant transcripts are produced in hei10-1. RT-PCR on flower buds from the hei10-1 mutant line or wild-type plants (Ws) are shown as well as the analyses of the hei10-1 RT-PCR products cloned and sequenced. B: Predicted HEI10 protein in wild type and hei10-1. Wild-type HEI10 protein sequence (in black) is compared to the predicted translational products issued of the cDNA variants described in A. (DOCX) [file pgen.1002799.s002.docx]

**Figure S2: Molecular characterisation of the *hei10-1* allele.**

**A: Aberrant transcripts are produced in *hei10-1***

**
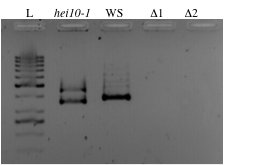
**

RT-PCR on flower buds from the *hei10-1* mutant line or wild-type plants (Ws).

*HEI10* amplification was obtained after two rounds of PCR, first with primers P21 and P5R (25 cycles at 65°C), and then with primers P21 and P10 (25 cycles at 65°C). The expected amplification size for wild-type cDNA sample is 374pb, and for genomic amplification is 834pb.

L : Fermentas 50pb DNA ladder

Δ1 : First PCR water control

Δ2 : Nested PCR water control

Sequences of the *hei10-1* RT-PCR products are analysed below.

The 5’ HEI10 genomic region is indicated (introns in black, exons in red, and start codon highlighted in yellow). The pink sequence indicates the corresponding genomic sequence in *hei10-1* mutant. A 36bp deletion was detected in the third exon of the gene just after the start codon for translation.

In **purple**, **orange** and **blue**: sequences of the RT-PCR clones ( P21+P10 primers on *hei10-1* cDNA).

P21

1760 • 1780 • 1800 • 1820 • 1840 • 1860 •

1751 TTGGTGATCTCAATGAGTTAGTTCAGTGGAGAATGGAAGTTTCATTTAGAGCATGGTTCTTCATCTTGTTTGATATGAAGTTAATTTGCAGGTGCGTAGCAGCTTGAGATACTTATATTAGAGGT 1875

|||||||||||||||||||||||||||||||||||||||||||||||||||||||||||||||||||||||||||||||||||||||||||||||||||||||||||||||||||||||||||||

TTGGTGATCTCAATGAGTTAGTTCAGTGGAGAATGGAAGTTTCATTTAGAGCATGGTTCTTCATCTTGTTTGATATGAAGTTAATTTGCAGGTGCGTAGCAGCTTGAGATACTTATATTAGAGGT

||||||||||||||||||||||||||||||||

1 =============================================================================================GCGTAGCAGCTTGAGATACTTATATTAGAGGT 32

• 20 •

||||||||||||||||||||||||||||||||

1 =============================================================================================GCGTAGCAGCTTGAGATACTTATATTAGAGGT 32

• 20 •

||||||||||||||||||||||||||||

1 =============================================================================================GCGTAGCAGCTTGAGATACTTATATTAG==== 28

• 20

1880 • 1900 • 1920 • 1940 • 1960 • 1980 • 2000

1876 TGACATGAGATGCAACGCGTGTTGGAGGGACCTGGAGGGCAGGGCCATTTCCACCACTTGCGGTCACTTATTATGTATCCTATTTACACTTTCTACTTTTCTTTTGCGTAGCAACACTTGTGAGC 2000

|||||||| ||| ||||| ||||||||||||||||||||||||||||||||||||||||||||||||||||||||||||||||||||||||

TGACATGAcATGag======gttgg============================cCACTTGCGGTCACTTATTATGTATCCTATTTACACTTTCTACTTTTCTTTTGCGTAGCAACACTTGTGAGC

|||||||| ||| ||||| ||||||||||||||||||||||||

33 TGACATGACATGAG======GTTGG============================CCACTTGCGGTCACTTATTATGTA================================================ 72

40 • 60 •

|||||||| ||| ||||| ||||||||||||||||||||||||||||||||||||||||||||||||||||||||||||||||||||||||

33 TGACATGACATGAG======GTTGG============================CCACTTGCGGTCACTTATTATGTATCCTATTTACACTTTCTACTTTTCTTTTGCGTAGCAACACTTGTGAGC 123

40 • 60 • 80 • 100 • 120

||||||||||||||||

29 =============================================================================================================AGCAACACTTGTGAGC 44

• 40

• 2020 • 2040 • 2060 • 2080 • 2100 • 2120

2001 TTGAATAAGATGACCTTAACACCATGAAAAGGTACTGAAGATGCCAGCAAGATTCTCAGTAATGATGGGGCATGTCCCATTTGTGATCAAGTACTCTCCAAGAGGTAAAACTTTCCTAGCCTAGC 2125

|||||||||||||||||||||||||||||||||||||||||||||||||||||||||||||||||||||||||||||||||||||||||||||||||||||||||||||||||||||||||||||

TTGAATAAGATGACCTTAACACCATGAAAAGGTACTGAAGATGCCAGCAAGATTCTCAGTAATGATGGGGCATGTCCCATTTGTGATCAAGTACTCTCCAAGAGGTAAAACTTTCCTAGCCTAGC

||||||||||||||||||||||||||||||||||||||||||||||||||||||||||||||||||||||

73 ==================================CTGAAGATGCCAGCAAGATTCTCAGTAATGATGGGGCATGTCCCATTTGTGATCAAGTACTCTCCAAGAG===================== 145

80 • 100 • 120 • 140

||||||||||||||||||||||||||||||||||||||||||||||||||||||||||||||||||||||||||||||||||||||||||||||||||||||||

124 TTGAATAAGATGACCTTAACACCATGAAAAGGTACTGAAGATGCCAGCAAGATTCTCAGTAATGATGGGGCATGTCCCATTTGTGATCAAGTACTCTCCAAGAG===================== 227

• 140 • 160 • 180 • 200 • 220

||||||||||||||||||||||||||||||||||||||||||||||||||||||||||||||||||||||||||||||||||||||||||||||||||||||||

45 TTGAATAAGATGACCTTAACACCATGAAAAGGTACTGAAGATGCCAGCAAGATTCTCAGTAATGATGGGGCATGTCCCATTTGTGATCAAGTACTCTCCAAGAG===================== 148

• 60 • 80 • 100 • 120 • 140

• 2140 • 2160 • 2180 • 2200 • 2220 • 2240 •

2126 TTCAGTTTAGCTCTAGTCGCCTTATTCTCCATTTGACAGTTAGTCTTTATCTTTCCATCTTAGTTTAATGAAACCTGTGGATATCAATCCAAATGAAGAATGGATAAATGTAAGTGGTTAAGCTG 2250

|||||||||||||||||||||||||||||||||||||||||||||||||||||||||||||||||||||||||||||||||||||||||||||||||||||||||||||||||||||||||||||

TTCAGTTTAGCTCTAGTCGCCTTATTCTCCATTTGACAGTTAGTCTTTATCTTTCCATCTTAGTTTAATGAAACCTGTGGATATCAATCCAAATGAAGAATGGATAAATGTAAGTGGTTAAGCTG

||||||||||||||||||||||||||||||||||||||||||||||

146 ===============================================================TTTAATGAAACCTGTGGATATCAATCCAAATGAAGAATGGATAAAT================ 191

• 160 • 180 •

||||||||||||||||||||||||||||||||||||||||||||||

228 ===============================================================TTTAATGAAACCTGTGGATATCAATCCAAATGAAGAATGGATAAAT================ 273

• 240 • 260 •

|||||||||||||||||||||||||||||||||||| |||||||||

149 ===============================================================TTTAATGAAACCTGTGGATATCAATCCAAATGAAGAGTGGATAAAT================ 194

• 160 • 180 •

2260 • 2280 • 2300 • 2320 • 2340 • 2360 •

2251 AAATACGCAAGGATACTATAGCTTTTCTTCTTTTCCAGTTTTCAATATTCCCATCTTACATCTCCTCGTTGCTTACATGTGTTATCAGATGGCGATGGCTGGAATTTCTCCACAAATACGTATCC 2375

|||||||||||||||||||||||||||||||||||||||||||||||||||||||||||||||||||||||||||||||||||||||||||||||||||||||||||||||||||||||||||||

AAATACGCAAGGATACTATAGCTTTTCTTCTTTTCCAGTTTTCAATATTCCCATCTTACATCTCCTCGTTGCTTACATGTGTTATCAGATGGCGATGGCTGGAATTTCTCCACAAATACGTATCC

|||||||||||||||||||||||||||||||

192 ========================================================================================ATGGCGATGGCTGGAATTTCTCCACAAATAC====== 222

200 • 220

|||||||||||||||||||||||||||||||

274 ========================================================================================ATGGCGATGGCTGGAATTTCTCCACAAATAC====== 304

280 • 300

|||||||||||||||||||||||||||||||

195 ========================================================================================ATGGCGATGGCTGGAATTTCTCCACAAATAC====== 225

200 • 220

2380 • 2400 • 2420 • 2440 • 2460 • 2480 • 2500

2376 TTTTCATAGATGTGCTCTTGGTAGAATTTTCAAGTTGTAACTATGGTTTTTCAATTGAAAATTGTCCCGCTGTATCTCAAATCTAAACTCAAGTGGTATAACTTTATCTCTTTTGCCTTTGTAAG 2500

|||||||||||||||||||||||||||||||||||||||||||||||||||||||||||||||||||||||||||||||||||||||||||||||||||||||||||||||||||||||||||||

TTTTCATAGATGTGCTCTTGGTAGAATTTTCAAGTTGTAACTATGGTTTTTCAATTGAAAATTGTCCCGCTGTATCTCAAATCTAAACTCAAGTGGTATAACTTTATCTCTTTTGCCTTTGTAAG

=============================================================================================================================

=============================================================================================================================

=============================================================================================================================

• 2520 • 2540 • 2560 • 2580 • 2600 • 2620

2501 AGTTGGTAAAACAACAGTTTAAGGTTCAAAAACCTCCTGTTTTTCTTAACTGAATCTAGTGATGAAGAGTGCATACCGAAGTGTAATGTTTTACATTGCCCAAAGAGACTTAGAGATGCAGTACA 2625

|||||||||||||||||||||||||||||||||||||||||||||||||||||||||||||||||||||||||||||||||||||||||||||||||||||||||||||||||||||||||||||

AGTTGGTAAAACAACAGTTTAAGGTTCAAAAACCTCCTGTTTTTCTTAACTGAATCTAGTGATGAAGAGTGCATACCGAAGTGTAATGTTTTACATTGCCCAAAGAGACTTAGAGATGCAGTACA

|||||||||||||||||||||||||||||||||||||||||||||||||||||||||||||

223 ================================================================AAGAGTGCATACCGAAGTGTAATGTTTTACATTGCCCAAAGAGACTTAGAGATGCAGTACA 283

• 240 • 260 • 280

||||||||||||||||||||||||||||||||||||||||||||||||||||||||||||||||||

305 ===========================================================TGATGAAGAGTGCATACCGAAGTGTAATGTTTTACATTGCCCAAAGAGACTTAGAGATGCAGTACA 370

• 320 • 340 • 360 •

||||||||||||||||||||||||||||||||||||||||||||||||||||||||||||||||||

226 ===========================================================TGATGAAGAGTGCATACCGAAGTGTAATGTTTTACATTGCCCAAAGAGACTTAGAGATGCAGTACA 291

• 240 • 260 • 280 •

P10

• 2640 • 2660 • 2680 • 2700 • 2720 • 2740 •

2626 AGATGAATAGAGTTGTTGCACAGTGTCGTCAGAAATGTGAGGGTATGCAAGCAAAGTTTAGCGAGAAAATGGAGCAGGTCCATACAGCATATCAGAAGATGGGCAAGAGGTGTCAGATGATGGAG 2750

|||||||||||||||||||||||||||||||||||||||||||||||||||||||||||||||||||||||||||||||||||||||||||||||||||||||||||||||||||||||||||||

AGATGAATAGAGTTGTTGCACAGTGTCGTCAGAAATGTGAGGGTATGCAAGCAAAGTTTAGCGAGAAAATGGAGCAGGTCCATACAGCATATCAGAAGATGGGCAAGAGGTGTCAGATGATGGAG

|||||||||||||||||||||||||||||||||||||||||||||||||||||

284 AGATGAATAGAGTTGTTGCACAGTGTCGTCAGAAATGTGAGGGTATGCAAGCA======================================================================== 336

• 300 • 320 •

|||||||||||||||||||||||||||||||||||||||||||||||||||||

371 AGATGAATAGAGTTGTTGCACAGTGTCGTCAGAAATGTGAGGGTATGCAAGCA======================================================================== 423

380 • 400 • 420

|||||||||||||||||||||||||||||||||||||||||||||||||||||

292 AGATGAATAGAGTTGTTGCACAGTGTCGTCAGAAATGTGAGGGTATGCAAGCA======================================================================== 344

300 • 320 • 340

**Figure S2B: Predicted HEI10 protein in wild type and *hei10-1*.**

Wild-type HEI10 protein sequence (in black) is compared to the predicted translational products issued of the cDNA variants described in A. The colour code is the same than on B panel.

1 -MRCNACWRDLEGRAISTTCGHLLCTEDASKILSNDGACPICDQVLSKSLM

MT*……………………………………………………………………………………………………………………………

MT*……………………………………………………………………………………………………………………………

MT*……………………………………………………………………………………………………………………………

MTLTP*……………………………………………………………………………………………………………………

51 –KPVDINPNEEWINMAMAGISPQILMKSAYRSVMFYIAQRDLEMQYKMNRV

……………………………………………………………………………………………………………………………………

……………………………………………………………………………………………………………………………………

……………………………………………………………………………………………………………………………………

……………………………………………………………………………………………………………………………………

101-VAQCRQKCEGMQAKFSEKMEQVHTAYQKMGKRCQMMEQEVENLTKDKQEL

……………………………………………………………………………………………………………………………………

……………………………………………………………………………………………………………………………………

……………………………………………………………………………………………………………………………………

……………………………………………………………………………………………………………………………………

151-QEKFSEKSRQKRKLDEMYDQLRSEYESVKRTAIQPANNFYPRHQEPDFFS

……………………………………………………………………………………………………………………………………

……………………………………………………………………………………………………………………………………

……………………………………………………………………………………………………………………………………

……………………………………………………………………………………………………………………………………

201-NPAVNMMENRETIRKDRSFFSPATPGPKDEIWPARQNSSNSGPFDISTDS

……………………………………………………………………………………………………………………………………

……………………………………………………………………………………………………………………………………

……………………………………………………………………………………………………………………………………

……………………………………………………………………………………………………………………………………

251-PAIPSDLGNRRAGRGHPVYGGGGTANPQSTLRNLILSPIKRSQLSRSRPQ

……………………………………………………………………………………………………………………………………

……………………………………………………………………………………………………………………………………

……………………………………………………………………………………………………………………………………

……………………………………………………………………………………………………………………………………

301-LFTL*

…………

…………

…………

…………
