# Supplementary material for: The Arabidopsis HEI10 Is a New ZMM Protein Related to Zip3
Source: PLoS Genet. 2012 Jul 26;8(7):e1002799. doi: 10.1371/journal.pgen.1002799 (PMC3405992; doi:10.1371/journal.pgen.1002799)

**Figure S3: *hei10-4* genomic region**

Schematic representation of At1g53490 genomic region as predicted in TAIR10. Amplification between primers 470P3 and 500-P1R generated a band in the *hei10-4* mutant only. Sequencing of this amplification product revealed that nucleotides 19,962,396 to19,971,065 are absent in *hei10-4*.

470P3 :GCAGTCCTGATATGCAAGATGCC

500P1R:GACCTCCACCAAGAGTTCCACC


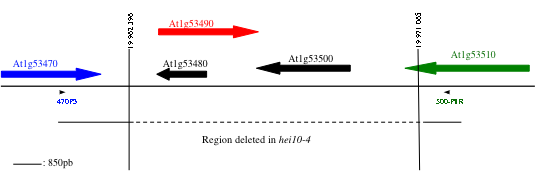

Supplement: Figure S3 — hei10-4 genomic region. Schematic representation of At1g53490 genomic region as predicted in TAIR10 (http://www.arabidopsis.org/). Amplification between primers 470P3 and 500-P1R generated a band in the hei10-4 mutant only. Sequencing of this amplification product revealed that nucleotides 19,962,396 to19,971,065 are absent in hei10-4. (DOCX) [file pgen.1002799.s003.docx]
