# Supplementary material for: The Arabidopsis HEI10 Is a New ZMM Protein Related to Zip3
Source: PLoS Genet. 2012 Jul 26;8(7):e1002799. doi: 10.1371/journal.pgen.1002799 (PMC3405992; doi:10.1371/journal.pgen.1002799)
Supplement: Figure S4 — hei10-5 genomic region. Schematic representation of At1g53490 genomic region as predicted in TAIR10 (http://www.arabidopsis.org/). Predicted ORF as well as primers used for hei10-5 deletion characterisation are shown. (DOCX) [file pgen.1002799.s004.docx]

**Figure S4: *hei10-5* genomic region.**

The diagram below is a schematic representation of At1g53490 genomic region as predicted in TAIR10 (http://www.arabidopsis.org/). Predicted ORF as well as primers used for *hei10-5* deletion characterisation are indicated and listed below. None of the primer combination indicated in the table provided amplification in *hei10-5* mutant showing that at least 13 kb are deleted in this mutant.


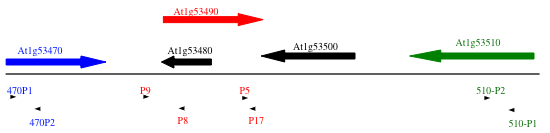


| **gene** | **primers** | **Position (Mb)** |
| --- | --- | --- |
| At1g53470 | 470P1 + 470P2 | 19,958,902 - 19,959,636 |
| At1g53490 | P9 + P8 | 19,962,583 - 19,963,755 |
| At1g53490 | P5 + P17 | 19,965,962 - 19,966,710 |
| At1g53510 | 510P2 + 510P1 | 19,972,946 - 19,973,843 |

470P1 : CCTCTGTCGTCAACGGCAGT

470P2: CACAAGCCCTAGCCATAGA

P9 :CATCCAACGTAGACTTGCAT

P8: TGGTAGTGGTGGCTCAGTGT

P5: GGAGCAGGTCCATACAGCA

P17:TACGTGAACAGCTGAGGGCG

510P2: GGGAGAGTTCAAGTCATCCCT

510P1: TGGAGTTGTGTGTGCAGCTA
