# Supplementary material for: The Arabidopsis HEI10 Is a New ZMM Protein Related to Zip3
Source: PLoS Genet. 2012 Jul 26;8(7):e1002799. doi: 10.1371/journal.pgen.1002799 (PMC3405992; doi:10.1371/journal.pgen.1002799)
Supplement: Figure S5 — HEI10 expression in different tissues. RT-PCR on cDNA isolated from leaves (Le), roots (R) and flower buds (B) of WS-4 wild-type plants were calibrated according to the expression of the phosphoribosyltransferase-encoding gene (APT, [84]) in A. They were then used to detect HEI10 expression after two rounds of nested PCR, first with primers P16 and P17, and second with primers P18 and P19. L: Fermentas 1 Kb DNA ladder. (DOCX) [file pgen.1002799.s005.docx]

**Figure S5: *HEI10* expression in different tissues.**

A

B


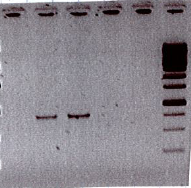


L Le R B Δ


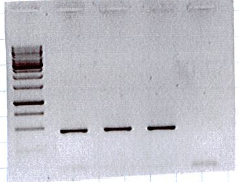

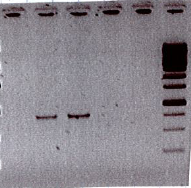

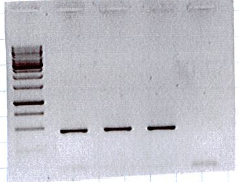


L Le R B Δ1 Δ2

RT-PCR on cDNA isolated from leaves (Le), roots (R) and flower buds (B) of WS-4 wild-type plants.

L : Fermentas 1Kb DNA ladder.

A : cDNA were calibrated according to the expression of the phosphoribosyltransferase-encoding gene (APT, Moffat et al. 1194, Gene. 143:211-216), after 30 amplification cycles at 60°C.

Δ : PCR water control.

B : *HEI10* amplification was obtained after two rounds of nested PCR, first with primers P16 and P17 (25 cycles at 65°C), and second with primers P18 and P19 (25 cycles at 65°C). Expected amplification size for wild-type cDNA sample : 730pb, and for genomic amplification : 1370pb.

Δ1 : First PCR water control

Δ 2 : Second PCR water control
