# Supplementary material for: The Arabidopsis HEI10 Is a New ZMM Protein Related to Zip3
Source: PLoS Genet. 2012 Jul 26;8(7):e1002799. doi: 10.1371/journal.pgen.1002799 (PMC3405992; doi:10.1371/journal.pgen.1002799)
Supplement: Figure S6 — hei10 mutants show low fertility and defects in male sporogenesis. A: Comparison of wild-type (Wt) and homozygous hei10-1 (hei10) mutant plants after a month in the greenhouse. Arrows show siliques that elongate in wild type but not in mutant. B: Meiotic products after anther clearing of wild type (wt) and hei10-1 mutant show abnormal male sporogenesis in hei10. (DOCX) [file pgen.1002799.s006.docx]

**Figure S6:** ***hei10* mutants show low fertility and defects in male sporogenesis.**

**A : *hei10* mutants are poorly fertile.**


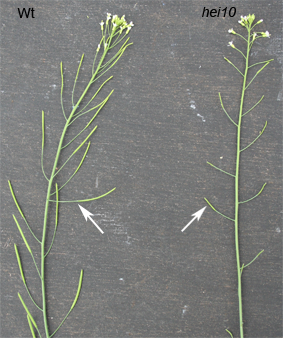


Comparison of wild-type (Wt) and homozygous *hei10-1* (*hei10*) mutant plants after a month in the greenhouse. Arrows show siliques that elongate in wild type but not in mutant.

***B : hei10 mutants show defects in male sporogenesis.***


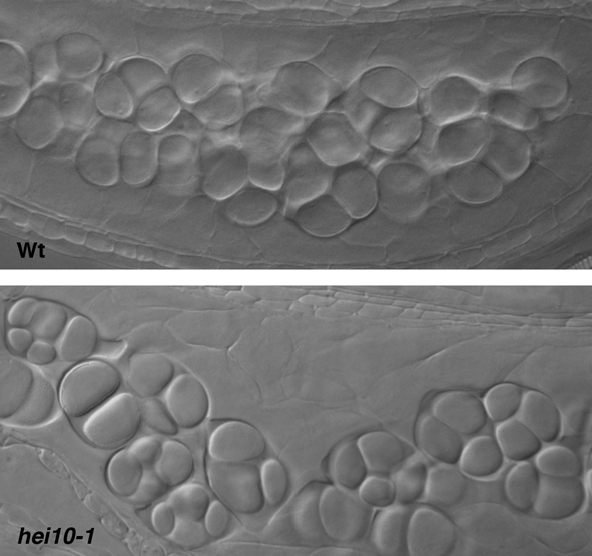


Meiotic products are shown after anther clearing for wild type (wt) and *hei10-1* mutant.
